# Navigating the Landscape of Coronary Microvascular Research: Trends, Triumphs, and Challenges Ahead

**DOI:** 10.31083/j.rcm2508288

**Published:** 2024-08-16

**Authors:** Yingyu Wang, Bing Wang, Hao Ling, Yuan Li, Sunjing Fu, Mengting Xu, Bingwei Li, Xueting Liu, Qin Wang, Ailing Li, Xu Zhang, Mingming Liu

**Affiliations:** ^1^Institute of Microcirculation, Chinese Academy of Medical Sciences & Peking Union Medical College, 100005 Beijing, China; ^2^International Center of Microvascular Medicine, Chinese Academy of Medical Sciences, 100005 Beijing, China; ^3^Department of Radiology, The Affiliated Changsha Central Hospital, Hengyang Medical School, University of South China, 410000 Changsha, Hunan, China; ^4^Laboratory of Electron Microscopy, Ultrastructural Pathology Center, Peking University First Hospital, 100005 Beijing, China; ^5^Diabetes Research Center, Chinese Academy of Medical Science, 100005 Beijing, China

**Keywords:** coronary microcirculation, coronary microvascular dysfunction, endothelial dysfunction, signaling pathways, traditional Chinese medicine

## Abstract

Coronary microvascular dysfunction (CMD) refers to structural and functional abnormalities of the microcirculation that impair myocardial perfusion. CMD plays a pivotal role in numerous cardiovascular diseases, including myocardial ischemia with non-obstructive coronary arteries, heart failure, and acute coronary syndromes. This review summarizes recent advances in CMD pathophysiology, assessment, and treatment strategies, as well as ongoing challenges and future research directions. Signaling pathways implicated in CMD pathogenesis include adenosine monophosphate-activated protein kinase/Krüppel-like factor 2/endothelial nitric oxide synthase (AMPK/KLF2/eNOS), nuclear factor erythroid 2-related factor 2/antioxidant response element (Nrf2/ARE), Angiotensin II (Ang II), endothelin-1 (ET-1), RhoA/Rho kinase, and insulin signaling. Dysregulation of these pathways leads to endothelial dysfunction, the hallmark of CMD. Treatment strategies aim to reduce myocardial oxygen demand, improve microcirculatory function, and restore endothelial homeostasis through mechanisms including vasodilation, anti-inflammation, and antioxidant effects. Traditional Chinese medicine (TCM) compounds exhibit therapeutic potential through multi-targeted actions. Small molecules and regenerative approaches offer precision therapies. However, challenges remain in translating findings to clinical practice and developing effective pharmacotherapies. Integration of engineering with medicine through microfabrication, tissue engineering and AI presents opportunities to advance the diagnosis, prediction, and treatment of CMD.

The landscape of coronary microvascular research, an area that has witnessed profound evolution over several decades, is a complex tapestry that researchers navigate with a mix of anticipation and caution. This transformation has been catalyzed by a confluence of technological advancements and innovative analytical methodologies. From the pioneering descriptive work of the 1960s outlining the rudimentary anatomy and physiology of coronary microvessels [1] to the recent surge in molecular explorations elucidating their pathophysiological roles in cardiovascular disease (CVD), our understanding of the coronary microvasculature has broadened dramatically.

Coronary microcirculation, a complex network of arterioles, capillaries, and venules, each with diameters under 500 µm, is pivotal to myocardial cell blood perfusion, oxygen distribution, and energy metabolism. Despite its significance, the intricate links between the structure and function of coronary microcirculation and CVD are not yet fully established. Consequently, the importance of coronary microcirculation is often overlooked or underestimated in cardiovascular clinical practice. Despite the new direction for CVD that coronary microcirculation offers, substantial challenges and unanswered questions linger. The processes underlying the microvascular contributions to various forms of CVD are still being unraveled. The translation of preclinical findings into clinical practice is frequently slow and fraught with difficulties. This review aims to offer a succinct summary of the microcirculatory mechanisms of coronary microvascular dysfunction (CMD) and its relationship with CVD, current treatment methods, and future prospects.

## 1. Coronary Microvascular Dysfunction

CMD represents a pathophysiological condition marked by the aberrant function of the coronary microvasculature. It is characterized by a constellation of disruptions in the coronary microcirculation, manifesting as an inadequate vasodilatory response to metabolic demand, heightened vasoconstrictive reactivity, and pathological remodeling of the microvascular architecture. CMD is precipitated by a multitude of pathogenic stimuli, leading to a heterogeneous set of mechanisms at play. These include but are not limited to, endothelium-dependent and -independent impairments in coronary vasodilation, augmented microvascular resistance due to structural alterations, and microvascular spasm. The presence of CMD signifies an early pathological state of cardiovascular disease and portends an adverse prognosis, underscoring its clinical significance as an early harbinger and potential therapeutic target in the continuum of cardiovascular pathology.

### 1.1 Structural and Functional Abnormality

CMD is acknowledged as a critical factor contributing to myocardial ischemia and angina pectoris. This condition arises from the intricate interplay of structural and functional abnormalities within the pre-coronary arterioles and small arteries. Notably, CMD is often implicated in the pathogenesis of ischemia with non-obstructive coronary arteries (INOCA), a clinical scenario where ischemic evidence is angiographically apparent despite the absence of significant coronary vessel disease [2]. The pathophysiological landscape of CMD is multifaceted, governed by a myriad of factors that dynamically evolve as the disease progresses.

The functional aspect of CMD is equally complex and reflects an interplay of impaired vasoreactivity, disrupted endothelial signaling, and altered metabolic responses. Vasomotor dysfunction, characterized by the inability of microvessels to adequately dilate in response to increased myocardial demand, contributes to an insufficient blood supply. This dysfunctional state is often attributed to a diminished bioavailability of vasodilators, and an upregulation of vasoconstrictive agents. Additionally, the endothelium’s role in anticoagulation and inflammation becomes compromised, further contributing to the pathology of CMD. Structurally, the coronary microvasculature exhibits marked alterations that are typified by the thickening of vessel walls, deformation of vascular lumens, and a reduction in capillary density. These changes are primarily a consequence of microvascular remodeling—a process triggered by early-stage CMD that disrupts microhemodynamics [3]. This remodeling is associated with increased microvascular resistance, and reduced perfusion capacity, which in turn predisposes the myocardium to ischemia and hypoxia. Furthermore, the microvascular bed is susceptible to damage manifesting as a paucity of microvessels, which is compounded by the increase of fibroblasts within the extracellular matrix and augmented synthesis of collagen. These events collectively precipitate microvascular fibrosis, culminating in a compromised microcirculatory function that can severely decrease myocardial vitality. The cumulative impact of these structural and functional derangements is a reduced coronary flow reserve (CFR), which signifies a diminished capacity of the coronary circulation to meet increased oxygen demands during stress.

**Fig. 1.**
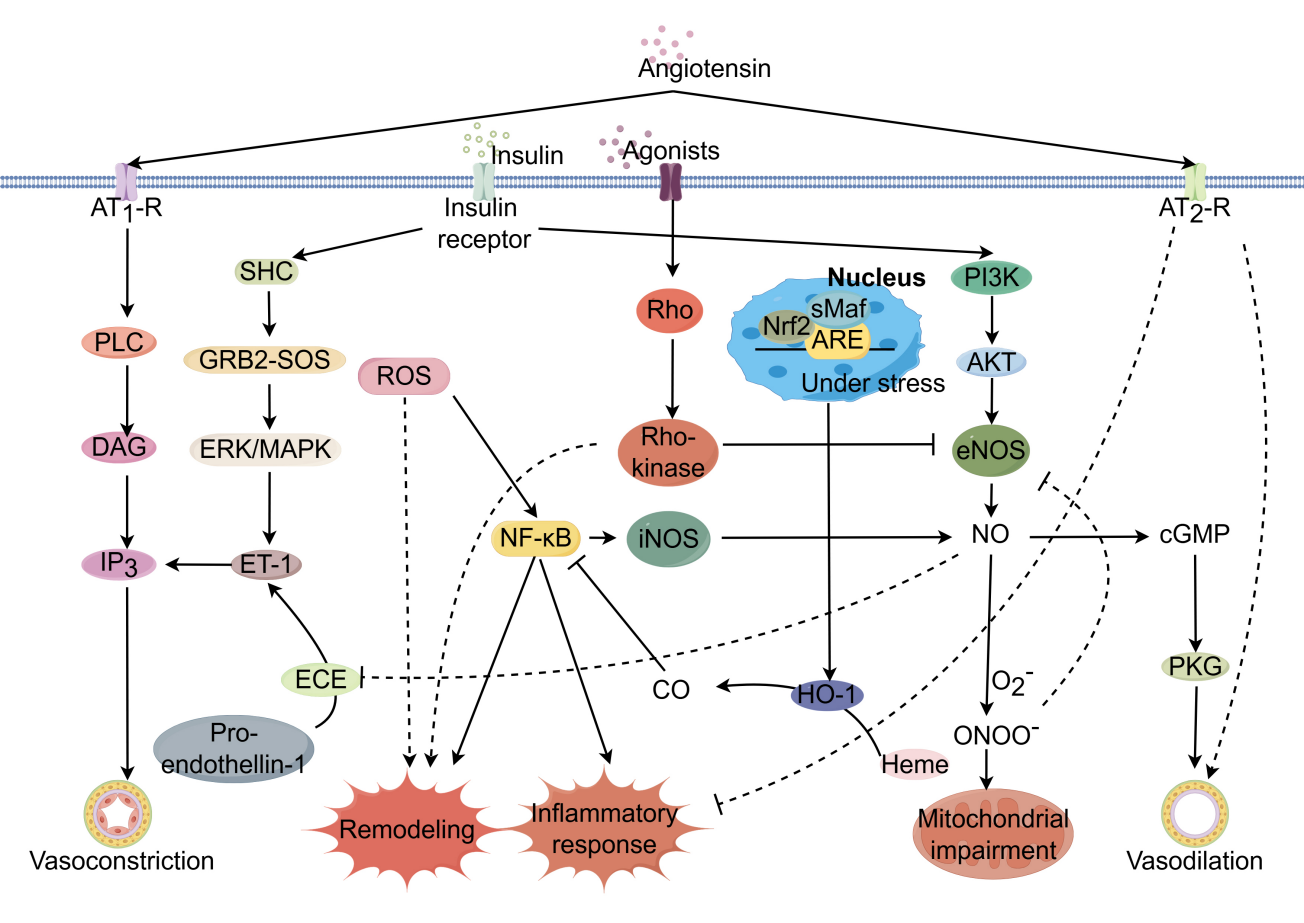
**Potential mechanisms regulating coronary microcirculation through modulation of signaling pathways**. Coronary microcirculatory function is governed by complex, interrelated signaling networks. Dysregulation of key pathways including AMPK/KLF2/eNOS, Nrf2/ARE, Ang II, ET-1, RhoA/Rho kinase, and insulin signaling can impair endothelial function and promote endothelial dysfunction, a hallmark of CMD. Activation of the AMPK/KLF2/eNOS axis increases eNOS phosphorylation and nitric oxide (NO) bioavailability, restoring vasodilation. Nrf2/ARE pathway induction suppresses oxidative stress and inflammation through the upregulation of antioxidant response genes. Inhibition of Ang II, ET-1, and RhoA/Rho kinase pathways decreases vasoconstriction by reducing downstream effectors like ET-1 and ROCK, improving endothelial function. PPARγ agonists and SGLT2 inhibitors enhance insulin sensitivity and glycemic control, mitigating insulin resistance-induced vasoconstriction and inflammation. Statins, PUFAs and plant polyphenols suppress inflammation through inhibition of NF-κB, the NLRP3 inflammasome and other pro-inflammatory mediators. Estrogen supplementation mimics estrogen’s impacts on angiogenesis, anti-inflammation and oxidative defense pathways to maintain endothelial homeostasis. Modulation of these signaling networks with pharmacological and nutritional interventions offers therapeutic potential for restoring the balance between vasodilation and vasoconstriction, thereby ameliorating CMD. Created using https://www.figdraw.com/. Abbreviations: AMPK, adenosine monophosphate-activated protein kinase; Ang, angiotensin; ARE, antioxidant response element; AT1R, angiotensin II type I receptor; cGMP, cyclic GMP; DAG, diacylglycerol; ECE, endothelin converting enzyme; eNOS, endothelial nitric oxide (NO) synthase; ERK, extracellular-regulated kinase; ET-1, endothelin1; GRB2, growth factor receptor binding protein-2; HO-1, heme oxygenase-1; iNOS, inducible nitric oxide synthase; IP3, inositol-1,4,5-triphosphate; MAPK, mitogen-activated protein kinase; NF-κB, nuclear factor kappa B; NO, nitric oxide; O2-, superoxide ion; PI3K, phosphatidylinositol3-kinase; PKG, cGMP-dependent protein kinase; PLC, phosphoinositide-specific phospholipase C; ROS, reactive oxygen species; SHC, Src (sarcoma) homology collagen-like-1; SOS, son of sevenless; ONOO-, peroxynitrite; ROCK, Rho-associated protein kinase; PPARγ, peroxisome proliferator-activated receptor alpha; PUFAs, polyunsaturated fatty acids; NLRP3, nucleotide-binding domain, leucine-rich repeat protein 3; Nrf2, nuclear factor erythroid 2-related factor 2; CMD, coronary microvascular dysfunction; SGLT2, sodium-glucose cotransporter 2; sMaf, small Maf proteins; CO, carbon oxide.

CMD involves a complex signaling cascade that involves a complex interplay of signaling pathways that impact vascular tone, inflammation, oxidative stress, and structural integrity (Fig. 1). This milieu fosters a fibrotic response, with increased deposition of extracellular matrix proteins such as collagen, leading to stiffening of the microvasculature and further functional compromise. While the classic signaling pathways, phosphatidylinositol 3-kinase (PI3K)/Akt (or PKB, protein kinase B) signaling pathway, and the nuclear factor kappa B (NF-κB) signaling pathway are implicated in cardiovascular health and disease and represent a significant area of study, our discussion of these pathways will be curtailed due to the constraints of this review. This review is specifically focused on delineating the signal transduction processes pertinent to endothelial cell function in coronary microcirculation.

### 1.2 Involved Signaling Pathways

#### 1.2.1 AMPK/KLF2/eNOS Signaling Pathway

The adenosine monophosphate-activated protein kinase/Krüppel-like factor 2/endothelial nitric oxide synthase (AMPK/KLF2/eNOS) signaling cascade is pivotal in maintaining endothelial integrity [4]. AMPK/KLF2 activation bolsters eNOS expression, thus facilitating nitric oxide (NO) synthesis, promoting vasorelaxation, and shielding against vascular inflammation and coagulation. Disruption of the AMPK/KLF2/eNOS axis has significant implications for the pathogenesis of CMD pathogenesis. Environmental stressors—hypoxia, inflammation, or oxidative stress—may impede AMPK activation, resulting in a decrease in KLF2 and eNOS levels [5]. The resultant NO shortfall hinders vasodilatory capacity, elevates vascular resistance, and diminishes coronary perfusion. Furthermore, reduced NO bioavailability lifts constraints on platelet aggregation and leukocyte adhesion, promoting a thrombogenic and inflammatory milieu [6]. This deleterious state inflicts collateral damage on the coronary microvasculature [7], fueling a vicious cycle of ischemia and exacerbating CMD progression (**Supplementary Fig. 1**).

#### 1.2.2 Nrf2/ARE Pathway

The nuclear factor erythroid 2-related factor 2/antioxidant response element (Nrf2/ARE) axis constitutes a principal cellular shield against oxidative stress. Oxidative stress enhances Nrf2 production and nuclear import, where it engages AREs within the promoters of genes encoding antioxidative armaments, initiating their transcription and subsequent combat against reactive oxygen species (ROS) [8]. This defense is crucial for preserving cellular equilibrium. Nevertheless, during sustained oxidative stress and inflammation, the Nrf2/ARE pathway may become compromised, culminating in an inadequate antioxidative response and the increase of ROS within the coronary microvasculature. Increasing ROS levels elicit oxidative injury to cellular macromolecules, potentially precipitating cellular dysfunction or apoptosis [9]. The subsequent endothelial dysfunction, resulting from increased ROS levels, compromises vasodilation, enhances vascular permeability, and promotes inflammatory and thrombotic states, thus exacerbating microvascular resistance, curtailing coronary perfusion, and provoking myocardial ischemia and microvascular remodeling (**Supplementary Fig. 2**).

#### 1.2.3 Angiotensin II Pathway

Angiotensin II (Ang II) signaling pathway is the key within the renin-angiotensin-aldosterone system (RAAS), orchestrating fluid homeostasis [10]. Pathologically elevated Ang II levels are implicated in the pathogenesis of CMD. Ang II operates through two principal receptors: the Ang II type 1 receptor (AT1R) and the Ang II type 2 receptor (AT2R). AT1R mediates vasoconstriction, aldosterone secretion, cellular proliferation, inflammation, and fibrotic processes [11]. Excessive AT1R activation disrupts the balance between vasodilatory and vasoconstrictive factors, characterized by increased vasoconstriction, diminished nitric oxide availability, and compromised coronary microvascular endothelial function, leading to reduced perfusion and ischemic manifestations. Ang II further triggers vascular inflammation, promoting vascular smooth muscle cell hypertrophy, hyperplasia, fibrosis, and luminal narrowing, thereby impeding microvascular adaptability to hemodynamic demands. Moreover, Ang II-induced ROS generation enhances oxidative stress, further deteriorating endothelial function and vascular inflammation (**Supplementary Fig. 3**).

#### 1.2.4 Endothelin-1 Signaling Pathway

Endothelin-1 (ET-1) critically modulates coronary microcirculatory dynamics through endothelin receptor type A (ETA) and endothelin receptor type B (ETB) receptors [12]. ETA receptors predominantly elicit vasoconstriction, whereas ETB receptors have dual roles, mediating both vasodilation and vasoconstriction. Excessive ET-1 signaling is associated with increased vasoconstriction, inflammation, and vascular remodeling, contributing to endothelial dysfunction [13]. Elevated ET-1 levels are indicative of endothelial dysfunction. Sustained pathway activation of this pathway promotes vascular remodeling and aggravates CMD [14]. ET-1 interaction with ETA receptors on vascular smooth muscle precipitates pronounced vasoconstriction, potentially leading to ischemic manifestations. This is compounded by ET-1-driven inflammatory responses and remodeling processes, including extracellular matrix deposition, which structurally compromises microvascular adaptability [15]. Furthermore, ET-1 may reduce NO bioavailability, exacerbating the imbalance between constrictive and vasodilatory forces within the coronary microcirculation (**Supplementary Fig. 4**).

#### 1.2.5 RhoA/Rho Kinase Pathway

The RhoA/Rho kinase axis is a critical regulator of coronary microvascular tone. Alterations in its activity have been implicated in cardiovascular pathogenesis [16]. This pathway governs cellular functions such as migration, proliferation, and apoptosis, and plays a pivotal role in modulating smooth muscle contractility and thereby vascular tone [17]. Under physiological conditions, RhoA/Rho kinase maintains basal vascular tone equilibrium. However, upon vasoconstrictive stimuli, RhoA activation stimulates Rho kinase, leading to increased myosin light chain phosphorylation, actin cytoskeleton reorganization, and smooth muscle contraction, resulting in enhanced vasoconstriction [18]. Pathological hyperactivation of this pathway contributes to endothelial dysfunction, vascular remodeling, and atherosclerosis, which are precursors to CMD [19]. Elevated RhoA/Rho kinase activity is associated with reduced NO synthesis and increased ET-1 production, thereby disrupting vasomotor balance. Clinically, enhanced Rho kinase activity correlates with impaired myocardial perfusion in coronary artery disease, independent of significant coronary artery stenosis [20] (**Supplementary Fig. 5**).

#### 1.2.6 Reactive Oxygen Species

ROS serve as signaling molecules crucial for cardiovascular homeostasis [21]. An imbalance marked by ROS overproduction or insufficient scavenging precipitates oxidative stress that is associated with microvascular dysfunction [22]. ROS influence coronary microvascular tone by modulating NO bioavailability. Excessive ROS react with NO to form peroxynitrite (ONOO-), diminishing NO levels, and thus promoting endothelial dysfunction characterized by impaired vasodilation and enhanced vasoconstriction [23]. This interplay is critical in the pathogenesis of CMD, where ROS-induced oxidative stress leads to endothelial apoptosis and mitochondrial dysregulation, contributing to myocardial ischemia-reperfusion injury. Moreover, ROS-mediated activation of matrix metalloproteinases (MMPs) drives vascular remodeling, with intimal thickening and fibrosis, narrowing the vascular lumen [24]. Endothelial cell apoptosis, triggered by ROS, disrupts the endothelial barrier, potentiating atherosclerotic lesion formation [25]. ROS also initiate an inflammatory response through the activation of NF-κB, escalating the transcription of pro-inflammatory cytokines and promoting leukocyte adhesion [26] (**Supplementary Fig. 6**).

#### 1.2.7 Insulin Signaling Pathway

Insulin orchestrates glucose homeostasis and influences vascular tone, with implications for coronary microcirculation [27]. Endothelial insulin signaling catalyzes NO production via the PI3K/Akt pathway, where insulin-mediated Akt phosphorylation activates eNOS, leading to vasodilation [28]. Conversely, insulin can promote vasoconstriction via the mitogen-activated protein kinases (MAPK) pathway by stimulating ET-1 production, and has both vasodilatory and vasoconstrictive properties which contributes to vascular homeostasis [29] (**Supplementary Fig. 7**). Insulin resistance often coexists with other cardiovascular risk factors, potentially exacerbating CMD. The pathway for glucose metabolism becomes less responsive, while the MAPK-dependent vasoconstrictive response may be preserved or amplified, altering the balance toward vasoconstriction, which can diminish coronary blood flow and contribute to CMD [30].

#### 1.2.8 Inflammatory Pathways

Inflammation plays a critical role in both the regulation and dysfunction of coronary microcirculation, with evidence implicating it in microvascular obstruction and contractility deficits, potentially resulting in micro-infarctions [31]. Systemic inflammatory states are known to induce functional disturbances within the coronary microvasculature, such as spasm and abnormal vasomotion, contributing to myocardial ischemia in the absence of large vessel obstruction [32]. The NF-κB signaling pathway is implicated in atherosclerotic processes [33]. Activated by various stressors, NF-κB modulates the transcription of pro-inflammatory genes, including those for adhesion molecules (vascular cell adhesion molecule-1, VCAM-1; intercellular cell adhesion molecule, ICAM-1) and cytokines (Interleukin-1, IL-1; Interleukin-1-6, IL-6; tumor necrosis factor, TNF-α), which stimulate leukocyte recruitment and adherence to endothelial cells, precipitating endothelial dysfunction [34]. The nucleotide-binding domain, leucine-rich repeat protein 3 (NLRP3) inflammasome, integral to inflammatory cytokine maturation, can be activated by damage-associated molecular patterns (DAMPs) or ROS, culminating in the release of IL-1β and IL-18, exacerbating endothelial damage and promoting vascular remodeling [35]. Similarly, the activation of the tumor necrosis factor (ligand) superfamily, member 18 (TNFSF18) inflammasome in coronary endothelial cells has been demonstrated to induce adhesion molecule expression, further perturbing coronary microcirculation in atherosclerosis [36]. Persistent activation of these inflammatory cascades can lead to microvascular remodeling, characterized by intimal thickening and luminal narrowing, which impairs coronary blood flow and can manifest as myocardial ischemia or infarction [37].

#### 1.2.9 Estrogen Signaling Pathway

Estrogen modulates cardiovascular function through interactions with estrogen receptors alpha (ERα) and beta (ERβ), which, upon estrogen binding, translocate to the nucleus and alter gene transcription responsible for vascular homeostasis [38]. Estrogen facilitates vasodilation, primarily by upregulating eNOS activity, increasing NO production [39], and attenuating vasoconstrictive responses to agents such as Ang II and norepinephrine [40]. Moreover, estrogen exerts anti-inflammatory effects, evidenced by downregulated expression of adhesion molecules and reduced cytokine-mediated leukocyte recruitment to the vascular wall [41]. Its antioxidative role is highlighted by the upregulation of antioxidant enzymes and the suppression of ROS formation and lipid peroxidation, thereby preserving endothelial function [42]. Estrogen also enhances mitochondrial efficiency in microvascular endothelial cells, optimizing calcium handling, adenosine triphosphate (ATP) production, and attenuating radical generation [43]—mechanisms that may underlie increased CMD susceptibility in postmenopausal women. Furthermore, estrogen promotes microvascular angiogenesis by upregulating pro-angiogenic factors such as vascular endothelial growth factor (VEGF) and basic fibroblast growth factor (bFGF), fostering new vessel formation and thus supporting microvascular integrity [44] (**Supplementary Fig. 8**). These multifaceted actions of estrogen are integral to the preservation of coronary microvascular health.

In the intricate matrix of CMD, the symphony of signaling pathways mentioned above play a pivotal role in dictating coronary health. These pathways, operating in a delicate balance, are integral to the regulation of endothelial function, which in turn governs the dynamics of vasomotion. Disruptions in this balance, through oxidative stress or inflammatory processes, lead to endothelial dysfunction and subsequent microvascular abnormalities characteristic of CMD. Central to CMD research is the understanding that no single signaling pathway operates in isolation. Instead, a complex interplay of vasodilatory and vasoconstrictive signals, coupled with metabolic and hormonal influences, contributes to the CMD landscape. Therapeutic strategies, therefore, must adopt a holistic approach, targeting the network of pathways to restore endothelial health and microvascular function.

## 2. Microcirculation Abnormalities and Coronary Artery Diseases

### 2.1 Coronary Microvascular Dysfunction and Coronary Artery Disease

CMD plays a pivotal role across a broad spectrum of cardiovascular diseases, from coronary artery disease (CAD) to various heart failure phenotypes and structural heart diseases (Table 1).

**Table 1.** **Role of coronary microvascular dysfunction in different cardiac/cardiovascular diseases**.

Different cardiac/cardiovascular diseases		Pathogenesis
Acute coronary syndromes	STEMI	Microvascular obstruction, ischemic-reperfusion injury, myocardial edema with microvascular compression, pre-existing CMD
		Plaque disruption/rupture and thromboembolism, microvascular spasm
Chronic coronary syndromes	Obstructive coronary artery disease	Microvascular remodeling, impaired capacity of maximal vasodilation
	INOCA	Microvascular spasm, impaired vasodilatory responses and fibrosis, chronic inflammation
Cardiomyopathy	HCM	Coronary microvascular remodeling, replacement fibrosis
Valvular heart disease	AS	Left ventricular hypertrophy, capillary rarefaction, arteriolar remodeling, perivascular fibrosis
Heart failure	HFpEF	Microvascular inflammation and rarefaction, cardiac fibrosis and cardiomyocyte hypertrophy
	HFrEF	Microvascular rarefaction, adverse remodeling, replacement fibrosis

Notes: STEMI, ST-segment elevation myocardial infarction; INOCA, ischemia with non-obstructive coronary arteries; HCM, hypertrophic cardiomyopathy; AS, aortic stenosis; HFpEF, heart failure with preserved ejection fraction; HFrEF, heart failure with reduced ejection fraction; CMD, coronary microvascular dysfunction.

CMD is increasingly recognized as a significant factor contributing to myocardial ischemia in CAD, independent of obstructive coronary artery lesions. The pathophysiological interplay between CMD and CAD involves endothelial dysfunction, smooth muscle cell dysregulation, and inflammatory processes, which are exacerbated by systemic risk factors. The interplay between CMD and CAD is a complex and dynamic relationship that has significant clinical implications. Emerging evidence suggests a bidirectional relationship wherein CMD may exacerbate the progression of epicardial coronary atherosclerosis. The compensatory upregulation of endothelium-derived hyperpolarizing factors, while crucial for vasodilation, may also contribute to vascular proliferation, inflammation, and heightened thrombotic potential, potentially accelerating atherosclerotic processes.

Acute ST-segment elevation myocardial infarction (STEMI) represents a clinical scenario where the interdependence of CMD and CAD is starkly evident. Despite successful reperfusion via percutaneous coronary intervention (PCI) in the majority of STEMI cases, more than half of these patients exhibit persistent myocardial microcirculatory deficits, indicative of CMD, primarily driven by ischemia-reperfusion injury [45]. The prognostic significance of CMD in the aftermath of STEMI cannot be overstated. CMD serves as an independent predictor of left ventricular remodeling and heart failure post-infarction. The long-term outcomes of STEMI patients, including mortality and heart failure, are intricately linked to the integrity of the microcirculation [46]. CMD is also a prevalent and critical factor in non-ST-segment elevation acute coronary syndromes, adding another layer of complexity to the management of these conditions [47]. The role of the microvasculature extends beyond the immediate post-infarct period, as it is a determinant of left ventricular remodeling and overall prognosis.

### 2.2 Coronary Microvascular Dysfunction and Ischemia with Non-Obstructive Coronary Arteries

The pathophysiology underlying INOCA remains incompletely elucidated. However, current research implicates CMD and/or epicardial coronary artery spasm as primary events of myocardial ischemia and INOCA, with microvascular angina (MVA) and vasospastic angina (VSA) representing the principal clinical phenotypes [48]. Recent investigations have shown that CMD co-exists in approximately 45–60% of patients with INOCA [49]. In the absence of epicardial coronary stenoses, CMD has been identified in up to 50% of chronic coronary syndrome presentations and up to 20% of acute coronary syndrome cases, with both scenarios demonstrating an elevated propensity for adverse clinical outcomes [50]. CMD compromises the myocardium’s capacity to augment coronary blood flow (CBF) in response to metabolic demand, resulting in suboptimal microcirculatory perfusion and direct cardiomyocyte injury. The presence of CMD has been linked to an increased incidence of adverse cardiovascular events [51]. A meta-analysis comprising 6631 patients suspected of INOCA revealed that those with CMD had a 3.93-fold increase in mortality and a 5.16-fold rise in the incidence of adverse cardiovascular events compared to those with normal coronary microvascular function [52]. The difference in the risk of cardiovascular events among INOCA patients can be largely attributed to differences in coronary microvascular function [53].

### 2.3 Coronary Microvascular Dysfunction and Heart Failure with Preserved Ejection Fraction

Heart failure with preserved ejection fraction (HFpEF) is defined by a left ventricular ejection fraction ≥50% and is characterized by prolonged left ventricular isovolumic relaxation times, augmented left ventricular stiffness, elevated left ventricular end-diastolic pressures, and decelerated left ventricular filling dynamics. Elucidating CMD-related factors in HFpEF is important to understand its pathogenesis and to refine clinical trials for CMD-centric HFpEF therapeutics. The pathophysiology of HFpEF is characterized by endothelial cell activation, contributing to myocardial remodeling. Recurrent microvascular infarctions precipitated by coronary microvascular ischemia lead to diffuse myocardial fibrosis, and increased left ventricular stiffness, resulting in diastolic dysfunction. The constellation of coronary microvascular ischemia, cardiomyocyte injury, and stiffness are emerging as integral components in the pathophysiology of HFpEF [54]. The patient-reported outcomes measurement information system (PROMIS)-HFpEF trial found a 75% incidence of CMD in HFpEF absent significant coronary artery stenosis, associating CMD with endothelial dysfunction and markers indicative of severe heart failure [55]. Concentric left ventricular remodeling, endothelial-to-mesenchymal transition, fibrosis, and myocardial-vascular stiffening are pivotal in propagating myocardial diastolic dysfunction and the genesis of HFpEF. The burgeoning incidence of HFpEF is now found in half of all heart failure cases and is associated with increased mortality and rehospitalization [56]. Emerging evidence posits HFpEF as an entity with microvascular abnormalities [57]. CMD has been implicated in the pathogenesis and progression of HFpEF, exerting a significant influence on adverse patient outcomes. Diastolic dysfunction attributable to CMD has been shown to predict a more than a fivefold escalation in HFpEF hospitalization risk [58]. CMD portends a dismal prognosis in patients with HFpEF over a one-year observational period [59].

### 2.4 Coronary Microvascular Dysfunction and Heart Failure with Reduced Ejection Fraction

The association between CMD and HFrEF is less established compared to its role in HFpEF, but the systemic nature of microvascular disease suggests a possible link. The evolution of left ventricular hypertrophy (LVH) secondary to arterial hypertension into the diverse spectrums of heart failure—including HFrEF—highlights the intricate pathways of cardiac remodeling and dysfunction. As delineated by Drazner [60], patients with LVH may transition to HFrEF through a ‘direct pathway’, which could occur in the context of a myocardial infarction or even in its absence. This progression underscores the multifaceted nature of heart failure where structural heart changes, ischemic injury, and CMD conspire to compromise systolic function. Evidence from small studies indicates that abnormal CFR is a prognostic marker for adverse outcomes in HFrEF [51]. A retrospective analysis involving 510 HFrEF patients undergoing myocardial perfusion positron emission tomography (PET) to quantify CFR showed that a lower CFR (≤1.65) doubled the risk of major cardiovascular events relative to a higher CFR (>1.65) [61]. When dissecting factors contributing to reduced CFR into structural CMD (diminished hyperemic flow) and functional CMD (increased resting flow), decreased hyperemic flow was identified as a significant risk factor, increasing the relative risk of death and heart failure progression by 3.5 times [62]. Recent registry data have indicated that both structural and functional CMD are substantial contributors to worsening clinical outcomes of HFrEF [63]. The mechanisms behind this elevated resting flow in reduced left ventricular ejection fraction (LVEF) remain elusive but may be associated with heightened left ventricular end-diastolic pressure and myocardial oxygen demand, driven by an increased heart rate and left ventricular mass.

### 2.5 Coronary Microvascular Dysfunction and Hypertrophic Cardiomyopathy/Aortic Stenosis

The asymmetrical hypertrophy within the ventricular myocardium is a discernible characteristic of hypertrophic cardiomyopathy (HCM). The prevalence of CMD within the HCM patient cohort is well-documented. In these patients, the hypertrophy of ventricular walls, the anarchic disposition of cardiomyocytes, and the accumulation of interstitial fibrosis precipitate deleterious remodeling of the microvasculature, which in turn, orchestrates myocardial ischemia [64]. CMD emerges as a pivotal pathological entity influencing the clinical trajectory of patients with cardiac hypertrophy. The constriction and occlusion of microvessels, coupled with the attenuation of neovascular formation, orchestrate a decreased energy supply to the hypertrophic cardiomyocytes. Over time, the relentless progression of CMD may incite recurrent episodes of myocardial ischemia and cardiomyocyte apoptosis, inexorably advancing towards the clinical denouement of heart failure and subsequent mortality. Clinically, the severity of CMD in HCM manifests as a spectrum of decline in CFR, inversely proportional to the extent of myocardial hypertrophy. Studies have shown a correlation between the severity of CMD and adverse prognostic outcomes in HCM patients; those presenting with more pronounced CMD are predisposed to an escalated risk of long-term morbidity and mortality [65]. Pathologically, significant arteriolar intimal proliferation and vessel wall hypertrophy have been observed in cardiac tissue from HCM patients, manifesting as marked vascular lumen stenosis. Cardiac magnetic resonance imaging further corroborates the prevalence of CMD-induced myocardial ischemia in approximately 50–80% of individuals diagnosed with HCM [66].

In patients with aortic stenosis (AS), CMD is intricately associated with the hemodynamic severity and ventricular responses to valvular obstruction. AS, defined by the latest guidelines [67], stratifies into normal-flow high-gradient and low-flow low-gradient phenotypes, each with distinct impacts on cardiac function and subsequent clinical management strategies. The pathophysiology of AS involves a complex interplay between endothelial disruption, inflammatory responses, progressive LVH, and concomitant microvascular impairment. This multifaceted cardiac remodeling results in a supply-demand mismatch, as evidenced by a significant decrease in subendocardial myocardial blood flow (MBF) and a reversal in the endocardial-epicardial blood flow ratio [68], which may lead to ischemia and angina despite unobstructed epicardial coronary arteries. After transcatheter aortic valve implantation (TAVI), there is an observable restoration in myocardial perfusion and microcirculatory function due to the alleviation of mechanical obstruction [69], although microvascular autoregulatory responses may remain suboptimal, evidenced by reduced CFR and myocardial perfusion reserve (MPR) [70].

In summary, the evaluation and management of CMD should be tailored to the specific cardiovascular disease, considering the unique pathophysiological mechanisms and clinical implications in each scenario. Emerging evidence underscores the importance of integrating CMD assessment into the routine diagnostic workup of patients with cardiovascular disease to optimize therapeutic strategies and improve clinical outcomes.

## 3. Coronary Microcirculation Assessment Techniques and Diagnosis

CMD represents a pathophysiological conundrum wherein myocardial ischemia occurs in the absence of overt epicardial CAD. The precise elucidation of CMD necessitates a multifaceted diagnostic approach that goes beyond the delineation of epicardial artery patency, focusing instead on the interplay of coronary microcirculation dynamics. The determination of coronary microcirculatory impairment can be diagnosed through either invasive or non-invasive techniques, as outlined in Table 2. European Society of Cardiology guidelines currently provide a class IIa recommendation for invasive assessment and class IIb recommendation for non-invasive assessment [71]. The optimal technique for evaluation depends on clinical scenario, patient characteristics, the presenting symptoms (acute vs. chronic) or comorbidities, and the necessity or feasibility for serial methods [72].

**Table 2.** **An overview of diagnostic modalities of CMD**.

Index	Methods	Pros	Cons	Diagnostic thresholds
Invasive				
TIMI flow	Coronary angiography	Simple and readily procedure	Semi-quantitative parameter	TIMI grading ≤2
TFC		Without additional contrast use	Insufficient sensitivity	TFC >25 frames
CFR	Intracoronary Doppler	Multiple assessment methods	Variations in resting hemodynamics	<2.0–2.5
	Intracoronary thermodilution	Predicts adverse outcomes	Influenced by epicardial coronary stenoses	
			Not microcirculation specific	
IMR	Bolus thermodilution	Specific to CMD	Inter- and intra-observer variability	≥25
		Predicts adverse outcomes	Operator-dependent	or ≥40 (severe)
		Extensively validated	Additional need for hyperemic agents	
HMR	Intracoronary Doppler	Independent of resting coronary flow	Requires adequate Doppler-based signals	≥2.0–2.5
		Predicts adverse outcomes	Influenced by epicardial stenoses	
MRR	Continuous thermodilution	Independent when measured by continuous coronary thermodilution	No optimal cut-off points due to novelty	No defined
	Bolus thermodilution	Specific to microvasculature		
	Intracoronary Doppler	Independent of myocardial mass		
IMR_angio_	Angiography derived	Pressure-wire-free tool	Requires further study	≥25
		Accessible to the non-interventional cardiac catheterisation laboratory	May be influenced by inter- and intra-observer	or ≥40 (severe)
			variability	
Non-invasive				
CFRV	TTDC	Widely available	Examiner dependent	<2.0
		Inexpensive	Limited to LAD region	
		No radiation exposure	Epicardial coronary stenoses prior exclusion	
MBF	MCE	Widely available	Examiner dependent	<2.0
		Good correlation with MBF by PET	Affecting quality	
		No radiation or radioactivity	Epicardial coronary stenoses prior exclusion	
CFR	PET	Gold standard for non-invasive modalities of CMD	Expensive	<2.0
		Global evaluation of microvascular function	Limited spatial resolution	
			Epicardial coronary stenoses prior exclusion	
MPRI	CMR	No radiation exposure	Time-consuming	<2.0
		MVO assessment	Poor patient compliance	
		Excellent spatial resolution	Epicardial coronary stenoses prior exclusion	
		Tissue characterization		
MPR	Dynamic CTP	Assessment of all coronary territories	Risk of kidney disease	<2.0
		Anatomic and functional evaluation	Radiation exposure	

Notes: CMD, coronary microvascular dysfunction; TIMI, thrombolysis in myocardial infarction; TFC, TIMI frame count; CFR, coronary flow reserve; IMR, index of microvascular resistance; HMR, hyperemic microvascular resistance; MRR, microvascular resistance reserve; CFRV, coronary flow reserve velocity; TTDC, transthoracic Doppler echocardiography; MBF, myocardial blood flow; LAD, left anterior descending; MCE, myocardial contrast echocardiography; PET, positron emission tomography; CMR, cardiac magnetic resonance; MVO, coronary microvascular obstruction; MPRI, myocardial perfusion reserve index; MPR, myocardial perfusion reserve; CTP, computed tomography perfusion.

### 3.1 Invasive Functional Testing

Invasive functional testing is pivotal for delineating CMD and enhancing risk stratification. The suite of indices encompasses technologies such as Doppler pressure guidewires and combined thermistor/pressure coronary guidewires. CFR estimates the dynamic capacity of the coronary circulation to undergo dilation thereby augmenting blood flow under conditions of maximal metabolic exigency. It serves as an indirect measure for the vasodilatory competence of the coronary microcirculation and can be ascertained using Doppler-tipped catheters or thermodilution techniques. However, its reliability may be compromised by the presence of epicardial coronary stenoses, variations in resting hemodynamics, and the basal level of coronary blood flow [73]. Contrastingly, the index of microvascular resistance (IMR) provides a focused estimation of the minimal quantifiable microvascular resistance, delivering more definitive quantitative insights that are not influenced by prevailing hemodynamic fluctuations. A cutting-edge innovation, the pressure-wire-free non-hyperemic angiography-derived index of microcirculatory resistance (NH-IMR_angio_), leverages computational fluid dynamics and sophisticated mathematical modeling to evaluate microvascular status, circumventing the need for intracoronary hardware or adenosine-induced hyperemia [74]. Hyperemic microvascular resistance (HMR) is quantified as the quotient of distal coronary pressure to mean flow velocity during maximal hyperemia. Both HMR and IMR provide independent evaluations of CMD. The choice between these indices hinges on the measurement modality: IMR is derived when thermodilution is employed, whereas HMR is ascertained via Doppler flow velocity assessments. A novel parameter, the microvascular resistance reserve (MRR), is tailored to determine the vasodilator reserve of the coronary microcirculation and is defined as the ratio of basal microvascular resistance to HMR [75]. The MRR has emerged as a stand-alone and robust prognosticator for major adverse cardiac events as well as target vessel failure over a 5-year observational period. This metric underscores the prognostic relevance of invasive functional testing in the serial management of patients with CMD [76].

### 3.2 Non-Invasive Investigating Methods

Non-invasive methodologies serve as an adjunctive diagnostic tool, with transthoracic Doppler echocardiography (TDE) enabling the assessment of coronary flow velocity reserve (CFVR) in the left anterior descending artery, albeit its application is frequently marred by operator dependency and inconsistent image acquisition [77]. Advanced imaging techniques such as PET and cardiac magnetic resonance imaging (CMR) offer superior spatial resolution and the ability to quantify myocardial blood flow both regionally and globally. PET, in particular, delivers quantitative perfusion metrics, while CMR is the modality of choice for delineating coronary microvascular obstruction [78]. Additionally, the advent of cadmium-zinc-telluride-single photon emission computed tomography (CZT-SPECT) could be a promising alternative for identifying microvascular dysfunction, presenting a viable and potentially more cost-effective alternative to cardiac PET [79].

Besides, the establishment of consensus-driven diagnostic cut-off values for CMD remains a pressing concern, as these are integral to the standardization of the diagnostic process. Given the variability of these thresholds across imaging modalities and patient cohorts, the aggregation of data from multicenter studies and the formulation of expert consensus guidelines are imperative for the refinement of CMD diagnostic criteria. Taken together, the comprehensive diagnosis of CMD is predicated on a sophisticated understanding of coronary microvascular pathophysiology, determined by the integration of both invasive and non-invasive diagnostic modalities. This diagnostic synergy is crucial for the demarcation of CMD from other heterogeneous cardiovascular pathologies, enabling the initiation of tailored therapeutic interventions.

## 4. Microcirculatory Treatment Strategies for Coronary Microvascular Dysfunction

Our understanding of CMD has significantly progressed over recent years, revealing its pivotal role in the onset and progression of diverse cardiovascular diseases. Despite these advances, the exploration of the therapeutic landscape remains a challenging endeavor due to the inherent complexity of the microcirculatory system and the multifaceted nature of CMD pathology. This review seeks to elucidate microcirculatory treatment strategies for CMD, subdivided into three broad categories: prevailing pharmacological interventions, traditional Chinese medicine (TCM), and emerging strategies encompassing small molecules and physiotherapy modalities.

### 4.1 Prevailing Pharmacological Interventions

The therapeutic strategy for CMD requires a comprehensive approach, incorporating the combined use of multiple drug classes (Table 3) to optimize coronary microvascular function and patient outcomes. Combination therapies, such as the co-administration of statins with (angiotensin-converting enzyme inhibitors) ACEIs or (angiotensin II receptor blocker) ARBs, have been reported to enhance endothelial cell function and overall microvascular health. This multifaceted pharmacotherapy, tailored to the specific needs of patients with INOCA, STEMI, or HFpEF, represents the forefront of individualized cardiovascular care.

**Table 3.** **Integrated pharmacological interventions in myocardial hemodynamic and oxygenic dynamics and contractility**.

Drug type	Representatives	Targeting sites	Mechanisms
Beta receptor blocker	Nebivolol, atenolol, carvedilol, metoprolol	Primarily through selective antagonism of β1-adrenergic receptor	Decrease myocardial oxygen demand by attenuating the sinoatrial node’s rate of discharge, thereby reducing heart rate (negative chronotropic effect).
			Diminish myocardial contractility (negative inotropic effect), leading to a reduction in cardiac output.
			Exhibit vasodilatory properties through β2-receptor blockade.
CCB	Diltiazem	(Cardiac) voltage-dependent L-type calcium channels	Reduce myocardial contractility, which lowers myocardial oxygen demand.
			Induce coronary vasodilation, improving oxygen delivery to the myocardium.
			Reduce the sinoatrial and atrioventricular node conduction velocity.
			Moderate vasodilation on peripheral vasculature, contributing to a reduction in blood pressure and afterload.
	Amlodipine	(Vascular smooth muscle) voltage-dependent L-type calcium channels	Moderate vasodilation on peripheral vasculature.
			Improve coronary blood flow and relief from myocardial ischemia without significant changes in heart rate or atrioventricular conduction.
			Prevent abrupt changes in hemodynamics.
ACEI	Quinapril, perindopril, ramipril	Angiotensin converting enzyme	Inhibit the conversion of angiotensin I to angiotensin II, leading to reduced vasoconstriction and aldosterone-mediated sodium and water retention, which in turn lowers blood pressure and decreases the overall workload.
			Prevent the binding of angiotensin II to its receptors of vascular smooth muscle.
			Facilitate vasodilation and promote natriuresis.
			Attenuate the remodeling processes in the heart and vasculature.
ARB	Losartan, valsartan, olmesartan	Angiotensin II type 1 receptor	Selectively block the binding of angiotensin II to the AT1 receptor, leading to the inhibition of vasoconstriction, aldosterone secretion, and the modification of cardiovascular structure.
			Induce vasodilation, reduce arterial pressure and afterload, which affects myocardial oxygen demand.
			Mitigate the retention of sodium and water indirectly through the inhibition of aldosterone, contributing to a reduction in preload and cardiac workload.
			Regression of myocardial hypertrophy and fibrosis, thus attenuating pathological cardiac remodeling.
Myocardial energy drug	Trimetazidine, ranolazine	pFOX	Inhibit the enzyme mitochondrial long-chain 3-ketoacyl CoA thiolase (pFOX).
			Attenuate the rate of fatty acid oxidation, these agents facilitate a metabolic shift towards the more oxygen-efficient glucose oxidation for ATP production, leading to a reduction in proton production and, consequently, a decrease in intracellular acidosis and calcium overload.
			Enhance the efficiency of myocardial energy production, thereby reducing myocardial oxygen demands.
Statins	Rosuvastatin, atorvastatin	HMG-CoA reductase enzyme	Catalyze the conversion of HMG-CoA to mevalonate and effectively reduces the endogenous cholesterol, leading to an upregulation of hepatic LDL receptors and an increased clearance of circulating LDL particles.
			Improve endothelial function, stabilize atherosclerotic plaques, reduce oxidative stress and inflammation.
Potassium channel activator	Nicorandil	Potassium channel	Stimulate ATP-sensitive potassium channels of vascular smooth muscle cells, induce vasodilation.
			Reduce venous return (preload) and systemic vascular resistance (afterload), which lead to a diminished myocardial workload and, therefore, myocardial oxygen demand.
			Promote anti-ischemic effects by improving coronary blood flow and reducing coronary vasospasm.
			Enhance myocardial oxygen supply-demand ratio, alleviating symptoms of angina pectoris.
Other drugs	Ivabradine	Sinoatrial node	Optimize the myocardial oxygen supply-demand balance through disparate mechanisms of action.
			Act as a vasodilatory agent by activating ATP-sensitive potassium channels.
			Exhibit a selective and specific inhibitory action on the pacemaker current in the sinoatrial node, leading to a prolongation of diastole, enhancing myocardial perfusion and reducing myocardial oxygen consumption.

Notes: CCB, calcium channel blocker; ACEI, angiotensin converting enzyme inhibitor; ARB, angiotensin receptor blocker; pFOX, partial fatty acid oxidation; HMG-CoA, hydroxymethylglutaryl-CoA; AT1, angiotensin II type 1; LDL, low-density lipoprotein; ATP, adenosine triphosphate.

In the therapeutic landscape of INOCA, a precise pharmacological approach tailored to the individual patient’s pathophysiology is crucial. Beta-blockers, including Nebivolol, Atenolol, Carvedilol, and Metoprolol, serve to diminish myocardial oxygen consumption, thus attenuating the symptomatic burden and enhancing functional capacity in INOCA [80]. Additionally, calcium channel blockers such as Benidipine, Diltiazem, and Amlodipine, exert their therapeutic effect by moderating myocardial contractility and improving coronary blood flow, thereby addressing the ischemic manifestations of INOCA. ACEIs and ARBs, including Quinapril, Perindopril, and Ramipril, in addition to reducing of myocardial oxygen demand, decrease vasoconstriction, thereby enhancing myocardial perfusion. Metabolic modulators such as Trimetazidine and Ranolazine act on the cellular energetic level, optimizing the myocardial oxygen supply-demand ratio and consequently aiding in the restoration of coronary microcirculatory function. Lipid-lowering agents, notably statins such as Rosuvastatin and Atorvastatin, are integral to the management of INOCA, not merely for their cholesterol-lowering effect but also for their capacity to augment endothelial function and mitigate vascular inflammation. This dual action serves to alleviate coronary microvascular dysfunction. Additionally, therapeutic agents like Nicorandil and Ivabradine target homocysteine-induced microvascular dysfunction, thus restoring the intricate imbalance between myocardial blood supply and demand. These agents are now considered as adjunctive, second-line therapy across various INOCA subtypes.

In STEMI patients, the therapeutic focus extends to include interventions that address coronary microvascular function. Agents such as tirofiban have been employed to minimize microembolism, while the purinergic receptor P2Y, G-protein coupled, 12 protein (P2Y12) inhibitor ticagrelor has demonstrated superior efficacy in enhancing microcirculation post-PCI compared with clopidogrel [81]. This superiority has been substantiated by a comparative meta-analysis [82] and complements other therapeutic measures including beta-blockers and lipid-lowering therapy. In a randomized trial, the early initiation of alirocumab, a proprotein convertase subtilisin/kexin type 9 (PCSK9) inhibitor, in conjunction with high-intensity statin therapy, precipitated a significant reduction in low-density lipoprotein (LDL) cholesterol levels post-primary PCI for STEMI [83]. Kinin, administered postoperatively at a low dose, has been shown to reduce microcirculation resistance index, thus potentially improving long-term outcomes for STEMI patients [84].

CMD is diagnosed in a significant proportion of patients with HFpEF, and its presence bears substantial prognostic implications [85]. The understanding that CMD is central to the pathology of HFpEF is increasingly supported [86]. Sex-specific triggers for CMD in HFpEF have been proposed, with inflammation posited as a dominant factor in men, while ventricular remodeling and fibrosis are preeminent in women [87]. Among the limited pharmacologic therapies effective for HFpEF, sodium-glucose cotransporter 2 (SGLT2) inhibitors have emerged as a promising drug class, with their use supported by a meta-analysis and recent guidelines [88]. Intriguingly, SGLT2 inhibition has been associated with enhanced macro- and microvascular endothelial functions [89], signifying that the microvasculature is a potential therapeutic target in HFpEF. Moreover, studies such as Prospective comparison of ARNI with ARB on Management Of heart failUre with preserved ejectioN fracTion (PARAMOUNT), Prospective comparison of ARNI with ARB Global Outcomes in heart failure with preserved ejection fraction (PARAGON-HF), Prospective comparison of ARni vs. comorbidity-Associated conventionaL therapy on QOL And eXercise capacity (PARALLAX), and PROspective study of biomarkers, symptom improvement, and VEntricular remodeling during Sacubitril/Valsartan therapy for Heart Failure (PROVE-HF) have elucidated the benefits of sacubitril/valsartan in patients with HFpEF stem from multifaceted mechanisms, including the reversal of cardiac remodeling and improvement of coronary microcirculation, rather than its direct antihypertensive effect [90].

### 4.2 Traditional Chinese Medicine

TCM offers a promising complementary approach to managing CMD. It is effective in relieving symptoms, enhancing coronary microcirculation, protecting vascular endothelial function, inhibiting inflammation, and improving lipid profiles. TCM injections, such as Shenfu, Danhong, Shuxuening, and Shuxuetong, have demonstrated positive effects in improving angina symptoms and in electrocardiograms. Jinqiang Zhu *et al*. [91], reported that Shenfu injection can exert endothelium-dependent vasodilatory effects through the NO-cyclic GMP (cGMP) pathway on the vascular endothelium by upregulating the expression of eNOS mRNA and protein. Ginkgo Damo injection improved scores in the thrombolysis in myocardial infarction (TIMI) blood flow grading system, an indicator of coronary blood flow. Additionally, Danhong and compound Salvia miltiorrhiza injections were reported to increase NO and decrease ET-1 levels. Other injections, like Shuxuetong, ligustrazine, and compound Salvia miltiorrhiza, decreased the levels of inflammatory markers such as high-sensitivity C-reactive protein (hs-CRP), TNF-α, and IL-6, indicating an anti-inflammatory effect. Moreover, Danhong injection was found to increase E-selectin levels and decrease thrombomodulin levels, suggesting a role in the regulation of endothelial function.

TCM compounds have also shown significant efficacy in CMD treatment. Gelanxinning capsule, the compound of Pueraria lobata, hawthorn extract, and gypenosides, inhibit inflammation and restore endothelial function in CAD, and were shown to improve CMD in a single-center, randomized control trial, making it a potentially effective drug in non-CAD patients suffering from angina [92]. Shexiang Tongxin Dropping Pill alleviates M1 macrophage polarization-induced inflammation and endothelial dysfunction against CMD via the Dectin-1/Syk/interferon regulatory factor 5 (IRF5) pathway. Dectin-1-associated M1 macrophage polarization might be developed as a novel target for ameliorating CMD [93]. TCM decoctions, including Liqi Huatan Huoxue formula and Huayu Fuyuan capsules, have shown efficacy in improving clinical symptoms, exercise tolerance, and quality of life in CMD patients. Huoxue Tongmai Yixin decoction and Qihong powder improved CFRs, an index of microvascular function, and TIMI blood flow grading. Several decoctions, such as Liqi Huatan Huoxue formula and Huayu Fuyuan capsules increased NO levels and decreased ET-1 levels, suggesting a vasodilatory effect. Furthermore, decoctions such as Yiqi Tongluo recipe improved flow-mediated dilation, a measure of endothelial function, and Danqi Tongmai capsules decreased plasma levels of ET-1, AngⅡ, and IL-6, indicating a possible anti-inflammatory role. Some decoctions, such as Wenyang Huoxue and Xuefu Zhuyu, also improved lipid profiles by decreasing triglycerides (TG), total cholesterol (TC), and LDL-C levels and increasing high-density lipoprotein cholesterol (HDL-C) levels. Q. Yu, *et al*. [94] suggested a treatment combination of proprietary Chinese medicine and conventional MVA enhances clinical efficacy and improves coronary microvascular function. Musk Tonic Heart Pills [95] can improve coronary microcirculation function after myocardial I/R. The Xiaorong Wenban prescription can not only effectively improve myocardial blood perfusion but also protects vascular endothelial function in CMD patients after unstable angina pectoris [96]. Qishen Yiqi dropping pills can safely relieve coronary microcirculation dysfunction in patients with INOCA [97]. A more comprehensive understanding of these therapeutic interventions and their effects on coronary microcirculation has been reviewed by Zhihua Yang *et al*. [98].

**Fig. 2.**
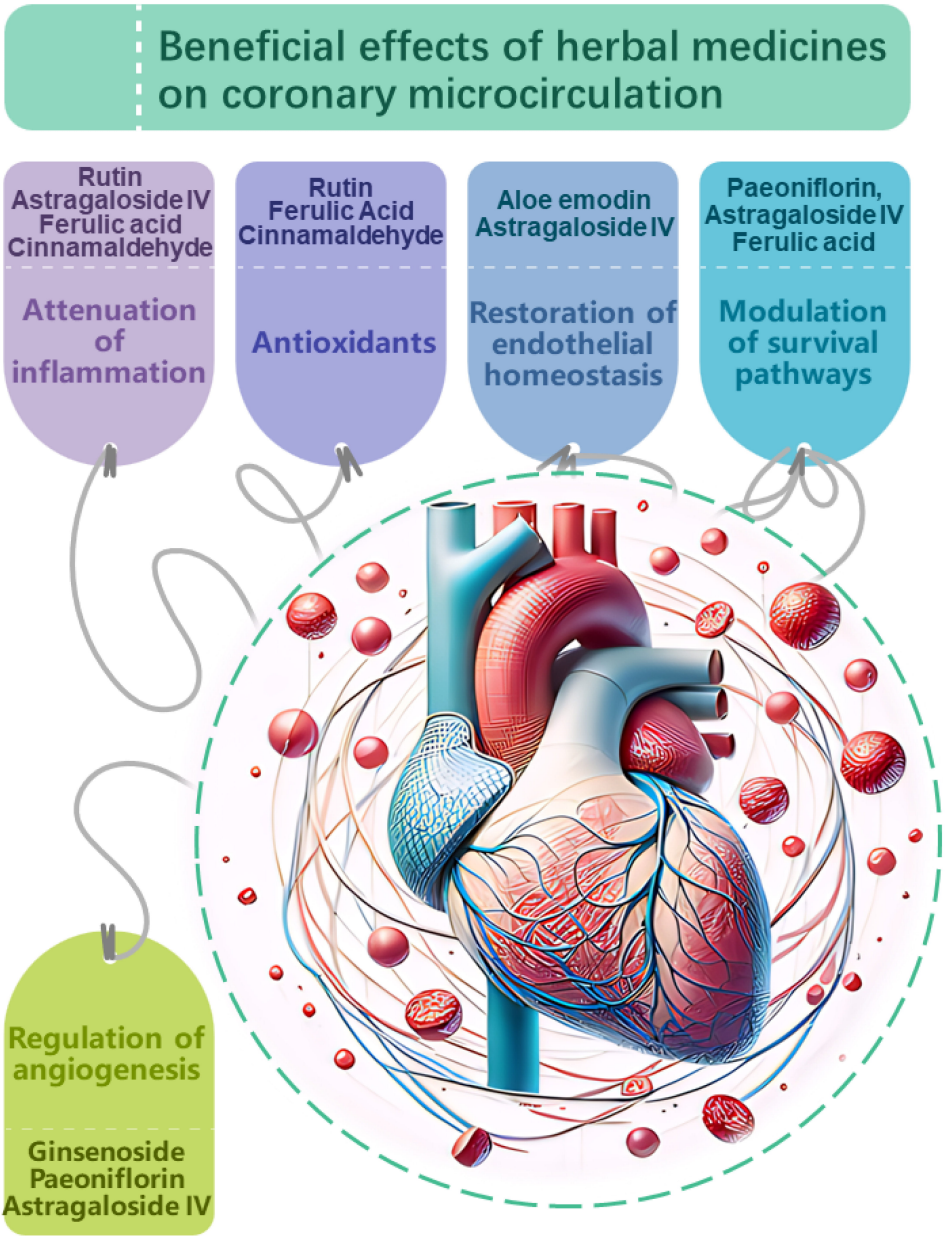
**Proposed mechanisms of action underlying the beneficial effects of herbal medicines on coronary microcirculation**. Herbal medicines have demonstrated potential in modulating coronary microcirculation through multi-faceted impacts on vascular physiology and pathology. Key targets include: Attenuation of inflammation. Compounds such as rutin, astragaloside IV, ferulic acid, and cinnamaldehyde suppress pro-inflammatory signaling and cytokine production, mitigating endothelial inflammation which compromises microvascular function. Antioxidant effects.Antioxidants, including rutin, ferulic acid and cinnamaldehyde neutralize oxidative stress, protecting the coronary microvasculature from damage induced by reactive oxygen species. Restoration of endothelial homeostasis. Compounds like aloe emodin and astragaloside IV stabilize endothelial junctions and barrier integrity, while ginsenoside, perillaldehyde and rutin enhance nitric oxide bioavailability, promoting vasodilation and balanced vascular tone.Modulation of cell survival pathways. Paeoniflorin, astragaloside IV and ferulic acid influence Akt/ERK and AMPK/TGF-β signaling to inhibit fibrosis, hypertrophy and apoptosis, preventing morphological deterioration in the microvascular bed. Regulation of angiogenesis. Ginsenoside, paeoniflorin and astragaloside IV boost angiogenic factor expression and microvessel formation, improving ischemic tolerance and nutritional perfusion. Through targeted actions on these molecular and cellular processes, herbal medicines may beneficially revise coronary microcirculatory dysfunction. AMPK, adenosine monophosphate-activated protein kinase; ERK, extracellular-signal regulated kinase; TGF-β, transforming growth factor beta; Akt, protein kinase B.

In addition to the complex herbal formulas commonly associated with TCM, the therapeutic application of individual herbs also forms an integral part of TCM’s holistic approach to health and wellness. The use of single herbs allows for targeted intervention, providing a clear understanding of the herb’s specific properties and actions. While multi-herbal formulas offer synergistic effects, single herbs ensure a focused treatment, making it easier to monitor and assess their impact and effectiveness (Fig. 2).

TCM compounds have demonstrated a multitude of mechanisms that intersect with the core pathophysiological processes of CMD. These compounds, including flavonoids like Rutin [99] and Quercetin [100], anthraquinones such as aloe emodin [100, 101] and Emodin [102, 103, 104], phenolic acids like Ferulic Acid [105, 106], monoterpenoids exemplified by Perillaldehyde [107, 108, 109], and various saponins such as Notoginsenoside [110] and Ginsenosides [111, 112], converge on key therapeutic targets. These targets comprise anti-inflammatory and antioxidative pathways, modulation of endothelial and smooth muscle cell function, and the preservation of myocardial viability and vascular integrity. These compounds exert their effects through intricate signaling pathways, including extracellular-regulated kinase (ERK)1/2, Akt, Nrf2/heme oxygenase-1 (HO-1), NF-κB, and eNOS [113, 114, 115, 116, 117, 118, 119, 120], which orchestrate cellular responses to oxidative stress, inflammation, and apoptotic processes.

The vasodilatory effects of TCM compounds, such as those evidenced by Paeonol [112], Notoginsenoside [121], and Tanshinone [122], align with the need to enhance coronary microcirculation - a fundamental abnormality in CMD. These effects are substantiated by their ability to regulate vascular smooth muscle tone, endorse endothelial function, and promote angiogenesis, thus facilitating blood flow and nutrient delivery to the myocardium. By harmonizing the multifaceted pharmacological actions of these compounds, such as mitigating endothelial dysfunction, suppressing myocardial hypertrophy, and fostering coronary microvascular integrity, a compelling case is made for their potential role in the holistic management of CMD. However, their clinical application warrants validation through rigorous trials to ensure efficacy and safety within the framework of integrated medicine.

Collectively, the pharmacological insights into both TCM compounds and single herbs reveal a convergence in pivotal cardioprotective mechanisms. These mechanisms encompass the attenuation of (pro-)inflammatory processes. Concurrently, these compounds exhibit potent antioxidative capabilities that protect against endothelial damage and preserve coronary microcirculation. Restoration of endothelial cell integrity, as well as enhancement of vasodilatory responses through the upregulation of eNOS and NO production, further characterize the therapeutic profile of these bioactive molecules. Additionally, protective effects against cardiomyocyte injury and the modulation of myocardial remodeling are achieved by influencing key signaling pathways that govern cell survival, fibrosis, and cardiac function. While the preclinical evidence underscores the promise of TCM compounds in the management of CMD, it is imperative that such findings are substantiated through rigorous clinical trials to ascertain their efficacy and safety in human populations, adhering to the standards of evidence-based medicine.

### 4.3 Small Molecules and Physiotherapy Modalities

While TCM offers potential therapeutic benefits for the management of CMD, the frontier of treatment options extends beyond the realm of these established practices. Emerging evidence accentuates the potential role of small molecules and regenerative medicine in addressing CMD. In contrast to the holistic approach of TCM, which often combines various natural ingredients, small molecules and regenerative therapies embody a more focused and targeted approach. This shift reflects a transition from the broad, systemic interventions typical of TCM, toward a more nuanced understanding of the pathophysiology of CMD. By honing in on specific cellular and molecular targets, these novel therapies promise a degree of precision and customization that could potentially revolutionize our approach to CMD management. Nevertheless, the integration of these two diverse approaches might yield the most effective strategy, incorporating the best of both worlds: the time-tested wisdom of TCM and the cutting-edge insights of modern molecular biology and regenerative medicine. The following section explores some of the emerging small molecule therapies and their potential role in the management of CMD.

The intricate landscape of CMD presents a compelling target for small molecule proteins [123], which offer a strategic approach to treat the delicate balance within the coronary microcirculation. Neuropeptide Y (NPY), has been implicated with augmenting CMD. Clinical studies have demonstrated that elevated levels of NPY in the coronary sinus correlate with microvascular constriction and subsequent diminished myocardial perfusion post-STEMI. This association underlies the potential of Y1 receptor antagonism as a therapeutic strategy, given the presence of Y1 receptors on vascular smooth muscle cells and their role in mediating NPY’s vasoconstrictive effects. Calpain, a calcium-activated cysteine protease, exemplifies a paradigm of functional duality within the coronary microvascular milieu, as evidenced by its intricate involvement in endothelial function modulation during ischemia-reperfusion therapy [124]. On the one hand, overactivation of calpain contributes to endothelial barrier disruption, exacerbating microvascular dysfunction by facilitating inflammatory and oxidative stress pathways, particularly in the setting of metabolic disturbances. Conversely, calpain’s proteolytic activity is essential for the removal of damaged proteins and for the facilitation of cellular repair mechanisms. This dichotomy necessitates a precision medicine approach in the therapeutic targeting of calpain, delicately balancing its inhibition to prevent acute microvascular damage while enabling its activity to support endothelial repair processes. The interplay between calpain and eNOS, further underscores the complexity of calpain’s role within the coronary microcirculation and the amelioration of CMD outcomes. The innovative compound TT-10 (TT-10 (TAZ-K) is an activator of YES-associated protein (YAP)-transcriptional enhancer factor domain (TEAD) activity), enriched with fluorine, also emerges as a novel entity, with preclinical studies suggesting its cardioprotective capabilities, including the promotion of cardiomyocyte proliferation and antiapoptotic actions. These small molecules highlight an expanding field, emphasizing targeted molecular interventions to alleviate CMD—a field poised for translation into clinical application to enhance coronary microcirculation and ameliorate outcomes related to myocardial ischemia.

The cellular therapies of Interest revolve around the role of myoblasts, hematopoietic stem cells (HSCs), endothelial progenitor cells (EPCs), and mesenchymal stem cells (MSCs) in enhancing cardiac function through improved microvasculature function. Skeletal myoblast transplantation has not proven effective in significantly improving left ventricular function, and has been associated with increased ventricular tachy-arrhythmias, raising safety concerns [125, 126]. Despite their ability to differentiate into a variety of blood lineage cells, HSCs (autologous CD34^+^ stem cells [127]) have not shown consistent benefits in cardiac function or in the ability to differentiate into cardiomyocytes post-transplantation. Their benefits have been attributed to paracrine angiogenic effects rather than direct myocardial repair of INOCA individuals suffering from refractory angina and CMD [128, 129, 130]. EPCs have been associated with stimulating microvascular network formation through both direct incorporation into blood vessels and via paracrine signaling, with clinical trials like Efficacy and Safety of Targeted Intramyocardial Delivery of Auto CD34+ Stem Cells for Improving Exercise Capacity in Subjects With Refractory Angina (RENEW) and Intramyocardial Transplantation of Bone Marrow Stem Cells in Addition to Coronary Artery Bypass Graft (CABG) Surgery (ClinicalTrials.gov Identifier: NCT00950274) (PERFECT) reporting improvements in microvascular perfusion and scar reduction, albeit without significant changes in ejection fraction [131, 132]. MSCs, on the other hand, have demonstrated cytoprotective and angiogenic effects, with preclinical studies showing increased peri-infarction angiogenesis and clinical trials such as Percutaneous Stem Cell Injection Delivery Effects on Neomyogenesis randomized trial (POSEIDON) and Prospective Randomized Study of Mesenchymal Stem Cell Therapy in Patients Undergoing Cardiac Surgery (PROMETHEUS) demonstrating reduced arrhythmias and improved left ventricular function after MSC transplantation [133, 134, 135]. This suggests that MSCs confer therapeutic benefits through microvascular enhancements and myocardial protection, contributing to functional recovery. While the direct myocardial integration of transplanted cells remains limited, the therapeutic potential of these cellular therapies may largely be attributed to their paracrine effects, which can promote angiogenesis and microcirculatory improvements, which are essential for cardiac repair and functional enhancement following myocardial injury.

Device-based therapies, such as intravascular lithotripsy (IVL) and percutaneous microsphere therapy, represent emergent modalities designed to ameliorate CMD through innovative mechanisms of action. IVL, an adaptation of shock wave therapy used to fracture calcific plaques in peripheral arteries, has been adapted for coronary application [136]. The principle underlying IVL involves the delivery of acoustic pressure waves that selectively disrupt calcified lesions while sparing soft tissue, thereby facilitating stent implantation and improving microvascular flow without the need for extensive tissue injury [137]. This technique, by virtue of its minimally invasive nature and specificity, holds promise for patients with calcific coronary artery disease, where traditional angioplasty may pose a risk of distal embolization and subsequent microvascular compromise. Percutaneous microsphere therapy is another frontier in device therapy, wherein biodegradable microspheres are delivered intra-arterially to promote microvascular perfusion. These microspheres can be loaded with therapeutic agents, such as growth factors or anti-inflammatory compounds, which are then locally released to enhance microcirculatory remodeling and repair [138]. Such targeted therapy could be particularly beneficial in CMD. Furthermore, the advent of bioresorbable vascular scaffolds (BVS) has introduced a new paradigm in the treatment of coronary artery disease. BVS are designed to provide temporary scaffolding to the vessel wall, ensuring luminal patency, and then they gradually dissolve, thereby reducing the risk of chronic vessel caging and preserving endothelial function [139]. The evolution of device therapies in coronary microcirculation is marked by the development of technologies that not only address macrovascular obstructions but also target the microvasculature with a level of precision that was previously unattainable. These advancements, coupled with a stratified management approach that integrates patient-specific risk factors and comorbidities, are poised to significantly improve the prognosis and quality of life for patients with CMD.

## 5. Conclusion and Future Perspectives

In the burgeoning field of coronary microvascular research, we have witnessed remarkable progress in delineating the pathophysiology of CMD and identifying potential therapeutic interventions. Despite these advances, the translation into clinical practice still has considerable obstacles, necessitating a concerted effort to refine diagnostic and therapeutic strategies. The advent of novel invasive coronary physiology indices has enhanced our diagnostic accuracy, yet their integration into routine clinical practice is impeded by a lack of consensus and standardized guidelines.

The “Clinical Coronary Microcirculation Function Assessment Workstation” is a state-of-the-art, interdisciplinary initiative, uniquely designed to tackle CMD with an integrated five-pronged approach. It combines Multimodality Modules—including intravascular ultrasound (IVUS), optical coherence tomography (OCT), CMR, and PET—to provide a comprehensive imaging assessment of the coronary microcirculation. This is complemented by a Multidimensional Analysis approach, employing computational fluid dynamics and advanced hemodynamics to decode the complexities of blood flow and vascular resistance. Central to the workstation is the Multiparametric Data Acquisition, which gathers a broad spectrum of cardiac parameters, facilitating a nuanced understanding of myocardial microcirculatory perfusion and oxygenation. The workstation transcends conventional diagnostics with its Whole-Heart Microcirculation Assessment, offering a global evaluation of the cardiac microcirculatory network, critical for pinpointing diffuse or localized microvascular impairments. Finally, the Multidisciplinary Collaboration at its foundation synergizes the expertise of cardiologists, imaging experts, microcirculation scientists, and bioengineers, ensuring the translation of intricate data into actionable clinical practice. This comprehensive and collaborative model promises to redefine CMD diagnosis and therapy, propelling cardiac care into a new era of precision and personalized medicine.

In summary, the future of coronary microvascular research is contingent upon ongoing innovation, interdisciplinary collaboration, and the swift application of research insights into clinical settings. It is through these concerted efforts that we can aspire to surmount the existing challenges and significantly enhance patient outcomes in CMD.

## Abbreviations

ACEI, angiotensin converting enzyme inhibitor; Akt, or PKB, protein kinase B; AMPK/KLF2/eNOS, adenosine monophosphate-activated protein kinase/Krüppel-like factor 2/endothelial nitric oxide synthase; Ang II, angiotensin II; ARB, angiotensin receptor blocker; AS, aortic stenosis; AT1, angiotensin II type 1; AT1R, ang II type 1 receptor; AT2R, ang II type 2 receptor; ATP, adenosine triphosphate; bFGF, basic fibroblast growth factor; BVS, bioresorbable vascular scaffolds; CBF, coronary blood flow; CCB, calcium channel blocker; CFR, coronary flow reserve; CFVR, coronary flow velocity reserve; CMD, coronary microvascular dysfunction; CMR, cardiac magnetic resonance imaging; CVD, cardiovascular disease; CZT-SPECT, cadmium-zinc-telluride-single photon emission computed tomography; EPCs, endothelial progenitor cells; ERα, estrogen receptors alpha; ERβ, estrogen receptors beta; ET-1, endothelin-1; HCM, hypertrophic cardiomyopathy; HDL-C, high-density lipoprotein cholesterol; HFpEF, heart failure with preserved ejection fraction; HFrEF, heart failure with reduced ejection fraction; HMG-CoA, hydroxymethylglutaryl-CoA; HMR, hyperemic Microvascular Resistance; HSCs, hematopoietic stem cells; ICAM-1, intercellular cell adhesion molecule; IL-1, interleukin-1; IL-6, interleukin-6; IMR, index of Microvascular Resistance; INOCA, ischemia with non-obstructive coronary arteries; INOCA, ischemia with non-obstructive coronary arteries; IVL, intravascular lithotripsy; LDL, low-density lipoprotein; LVH, left ventricular hypertrophy; MBF, myocardial blood flow; MMPs, matrix metalloproteinases; MPR, myocardial perfusion reserve; MRR, microvascular Resistance Reserve; MSCs, mesenchymal stem cells; MVA, microvascular angina; NF-κB, nuclear factor kappa B; NH-IMR_angio_, non-hyperemic angiography-derived index of microcirculatory resistance; NPY, neuropeptide Y; Nrf2/ARE, nuclear factor erythroid 2-related factor 2/antioxidant response element; ONOO-, peroxynitrite; PCI, percutaneous coronary intervention; PET, positron emission tomography; pFOX, partial fatty acid oxidation; PI3K, phosphatidylinositol 3-kinase; RAAS, renin-angiotensin-aldosterone system; ROS, reactive oxygen species; SGLT2, sodium-glucose cotransporter 2; STEMI, ST-segment elevation myocardial infarction; TAVI, transcatheter aortic valve implantation; TC, total cholesterol; TCM, traditional Chinese medicine; TDE, transthoracic Doppler echocardiography; TG, triglycerides; TIMI, thrombolysis in myocardial infarction; TNF-α, tumor necrosis factor; VCAM-1, vascular cell adhesion molecule-1; VEGF, vascular endothelial growth factor; VSA, vasospastic angina.

## Supplementary Material

Supplementary material associated with this article can be found, in the online version, at https://doi.org/10.31083/j.rcm2508288.
