# Supplementary material for: Navigating the Landscape of Coronary Microvascular Research: Trends, Triumphs, and Challenges Ahead
Source: Rev Cardiovasc Med. 2024 Aug 16;25(8):288. doi: 10.31083/j.rcm2508288 (PMC11366996; doi:10.31083/j.rcm2508288)
Supplement: Supplementary file 1 [file 2153-8174-25-8-288-s1.docx]

**Navigating the Landscape of Coronary Microvascular Research: Trends, Triumphs, and Challenges Ahead**

**Supplementary Information**

**
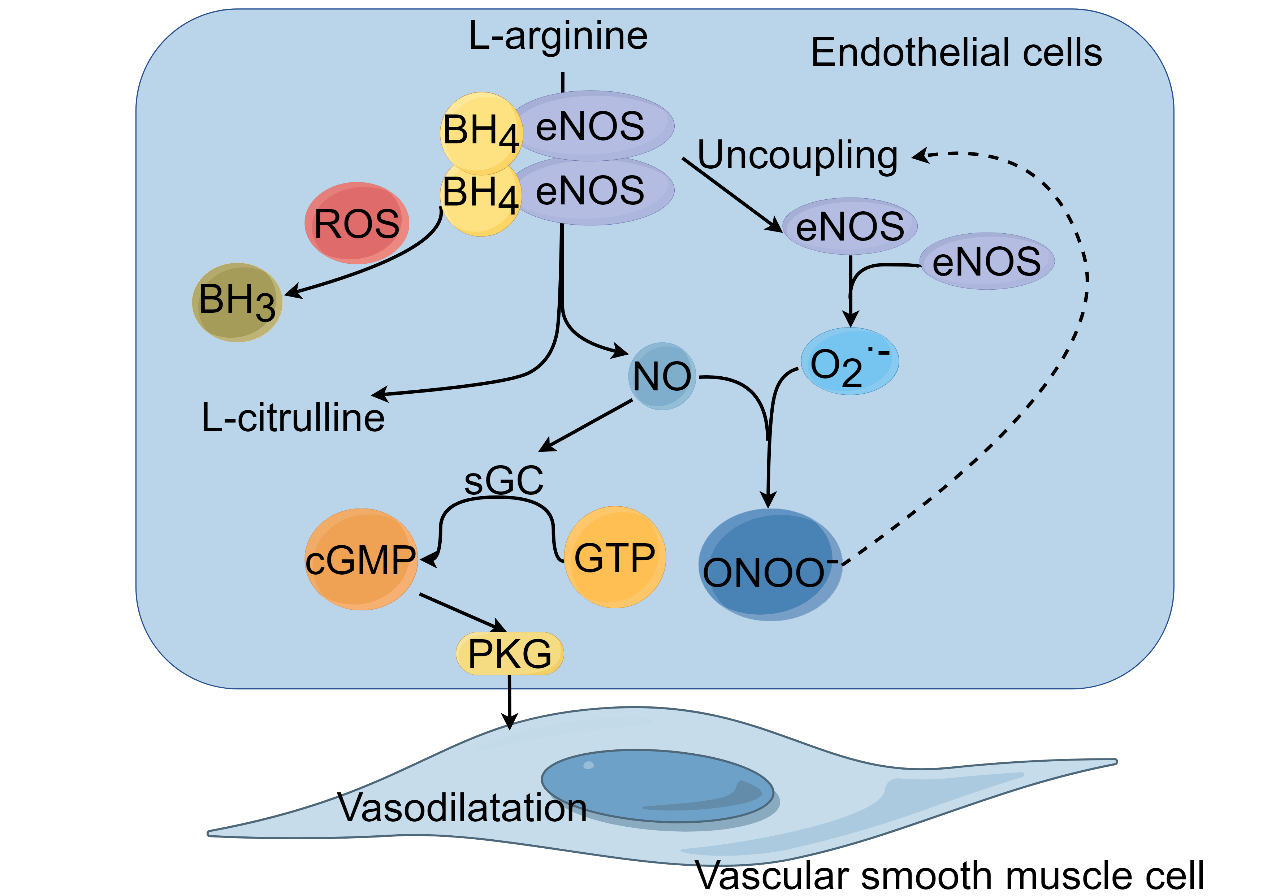
**

**Supplementary Fig. 1. AMPK/KLF2/eNOS signaling pathway.** AMPK/KLF2 activation bolsters eNOS expression, thus facilitating NO synthesis, promoting vasorelaxation, and shielding against vascular inflammation and coagulation. Environmental stressors—hypoxia, inflammation, or oxidative stress—may impede AMPK activation, with consequent detriments, including a decrease in KLF2 and eNOS levels. The resultant NO shortfall hinders vasodilatory capacity, elevates vascular resistance, and diminishes coronary perfusion. Furthermore, reduced NO bioavailability lifts constraints on platelet aggregation and leukocyte adhesion, promoting a thrombogenic and inflammatory milieu.

**
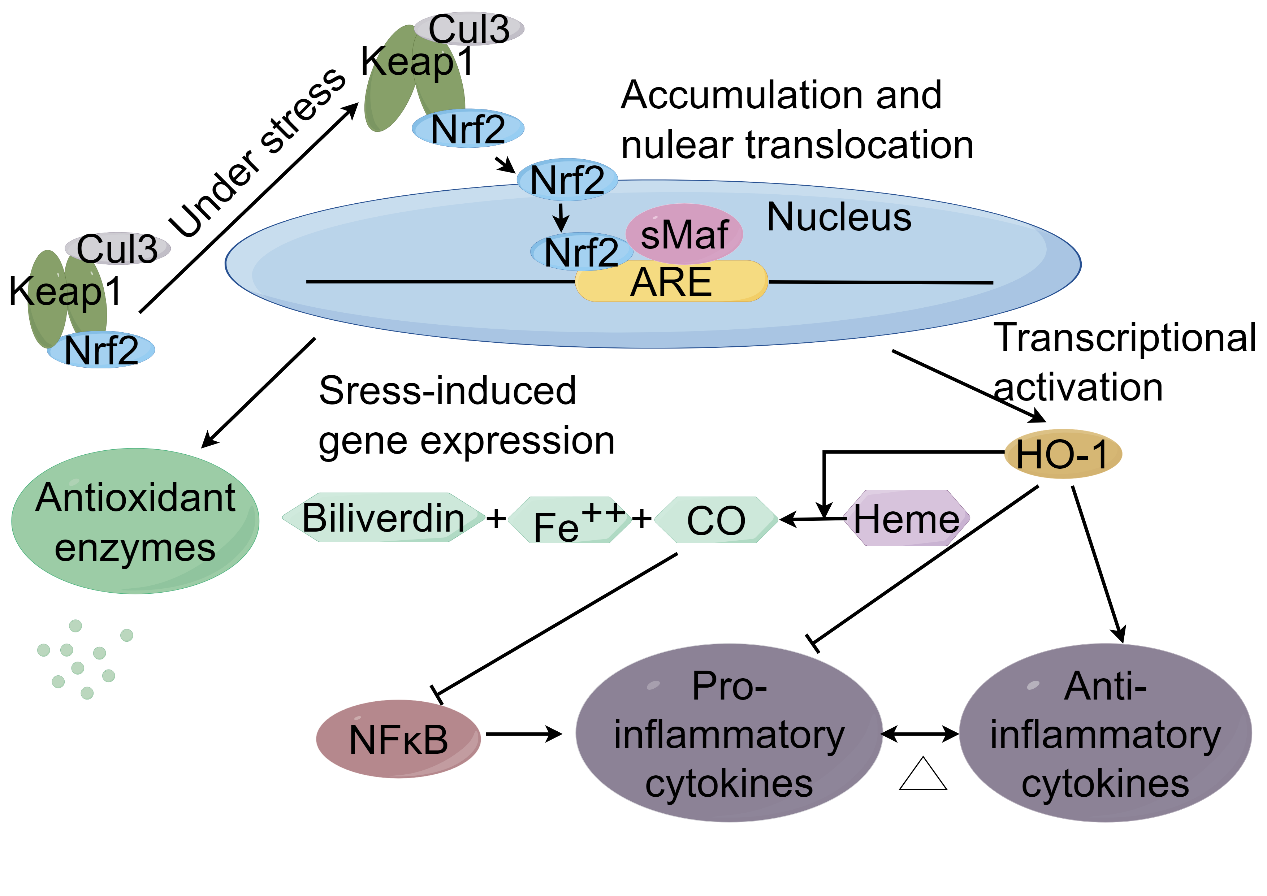
**

**Supplementary Fig. 2. Nrf2/ARE pathway.** Oxidative stress prompts Nrf2 liberation and nuclear import, where it engages AREs within the promoters of genes encoding antioxidative armaments, initiating their transcription and subsequent combat against ROS. During sustained oxidative stress and inflammation, the Nrf2/ARE pathway may become compromised, culminating in an inadequate antioxidative response and ROS accrual within the coronary microvasculature. Escalating ROS levels elicit oxidative harm to cellular macromolecules, potentially precipitating cellular dysfunction or apoptosis. Endothelial dysfunction, resultant from this oxidative barrage, compromises vasodilation, enhances vascular permeability, and promotes inflammatory and thrombotic states, thus exacerbating microvascular resistance.

**
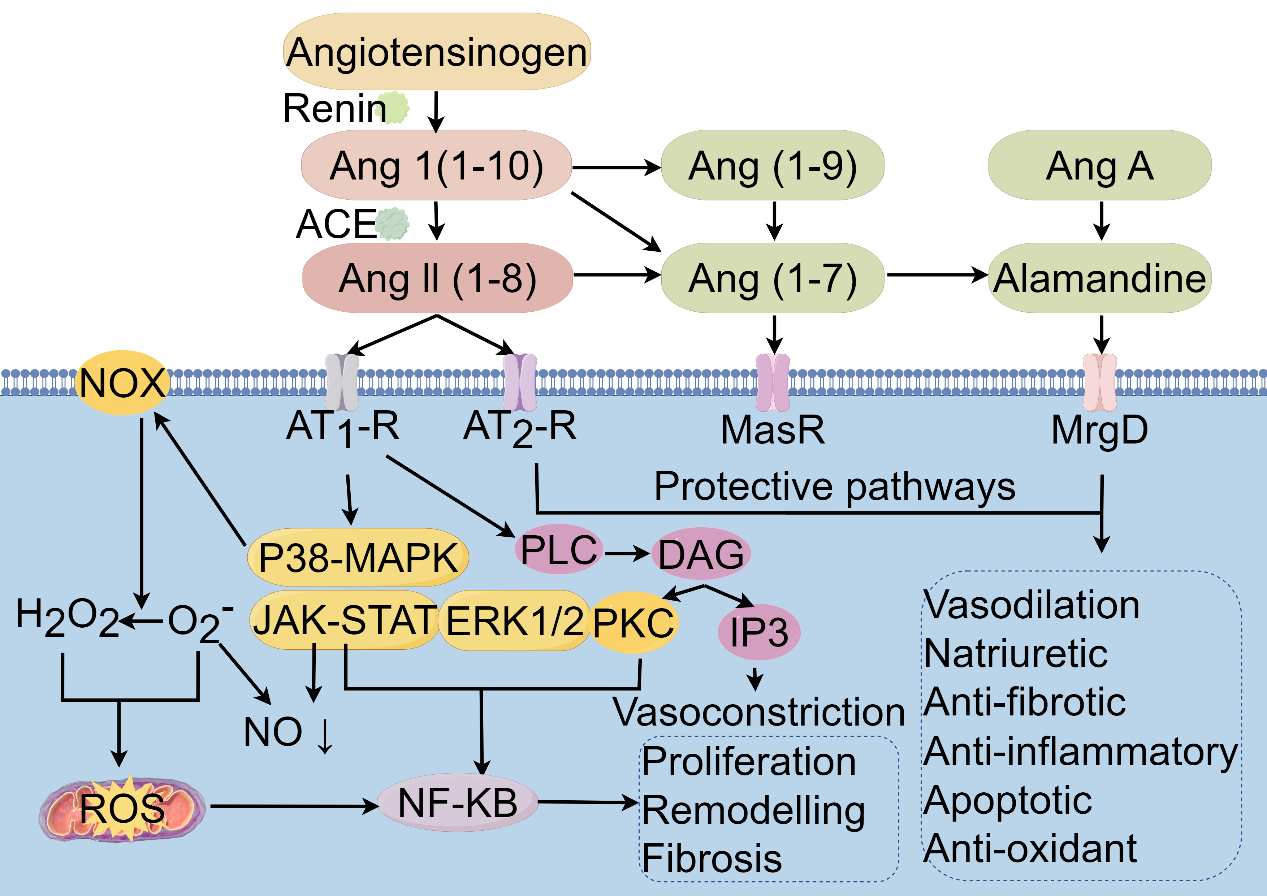
**

**Supplementary Fig. 3. Angiotensin II Pathway.** Ang II operates through two principal receptors: the Ang II type 1 receptor (AT1R) and the Ang II type 2 receptor (AT2R). AT1R mediates vasoconstriction, aldosterone secretion, cellular proliferation, inflammation, and fibrotic processes. Excessive AT1R activation disrupts the balance between vasodilatory and vasoconstrictive factors, characterized by increased vasoconstriction, diminished nitric oxide availability, and compromised coronary microvascular endothelial function, leading to reduced perfusion and ischemic manifestations. Ang II further triggers vascular inflammation, promoting vascular smooth muscle cell hypertrophy, hyperplasia, fibrosis, and luminal narrowing, impeding microvascular adaptability to hemodynamic demands. Ang II-induced ROS generation enhances oxidative stress, further deteriorating endothelial function and vascular inflammation.

**
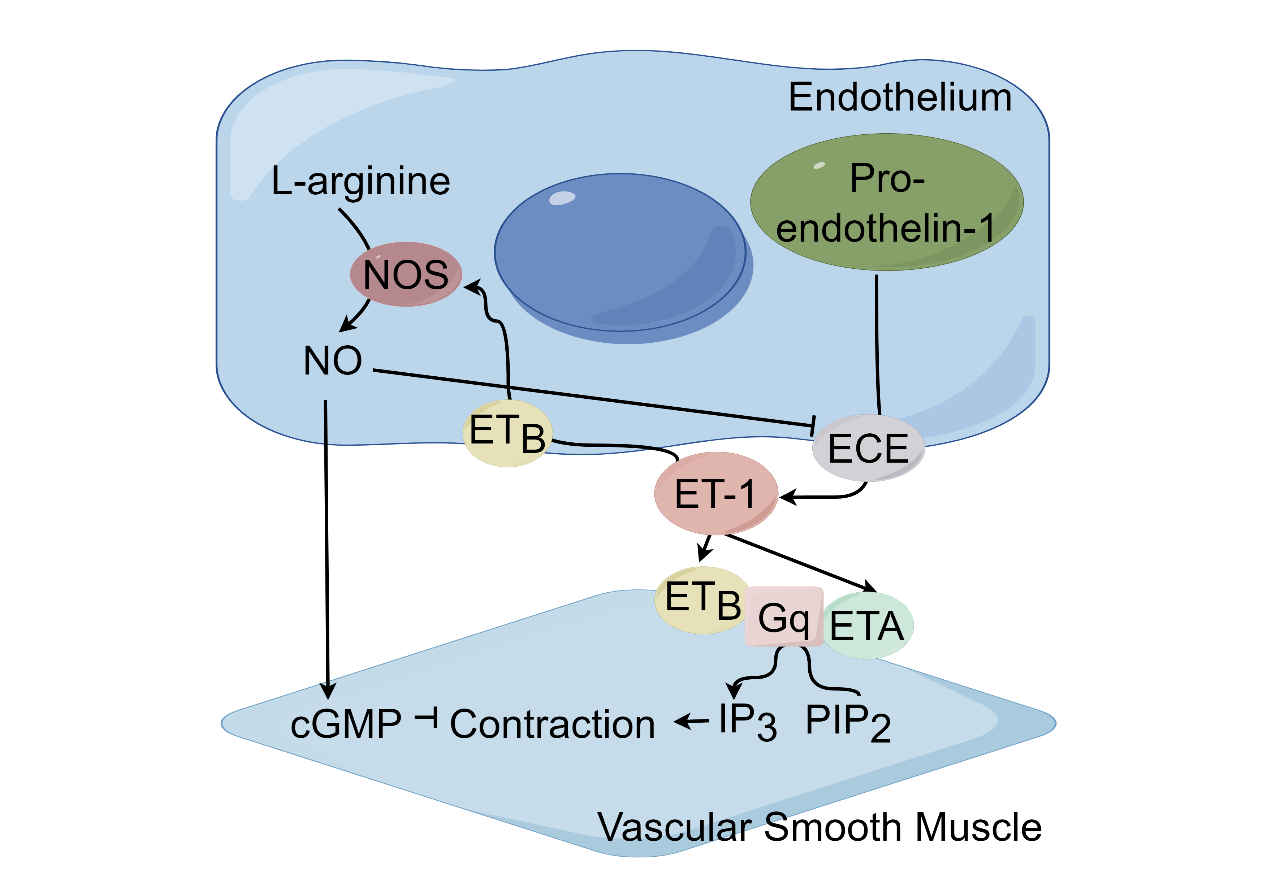
**

**Supplementary Fig. 4. Endothelin-1 signaling pathway.** Endothelin-1 (ET-1) critically modulates coronary microcirculatory dynamics through ETA and ETB receptors. ETA receptors predominantly elicit vasoconstriction, whereas ETB receptors have dual roles, mediating both vasodilation and vasoconstriction. Excessive ET-1 signaling is associated with increased vasoconstriction, inflammation, and vascular remodeling, contributing to endothelial dysfunction. Elevated ET-1 levels are indicative of endothelial perturbation, with its sustained pathway activation promoting vascular remodeling and aggravating CMD. ET-1 interaction with ETA receptors on vascular smooth muscle precipitates pronounced vasoconstriction, potentially leading to ischemic manifestations. This is compounded by ET-1-driven inflammatory responses and remodeling processes, including extracellular matrix deposition, which structurally compromise microvascular adaptability. Furthermore, ET-1 may reduce NO bioavailability, exacerbating the imbalance between constrictive and dilatory forces within the coronary microcirculation.

**
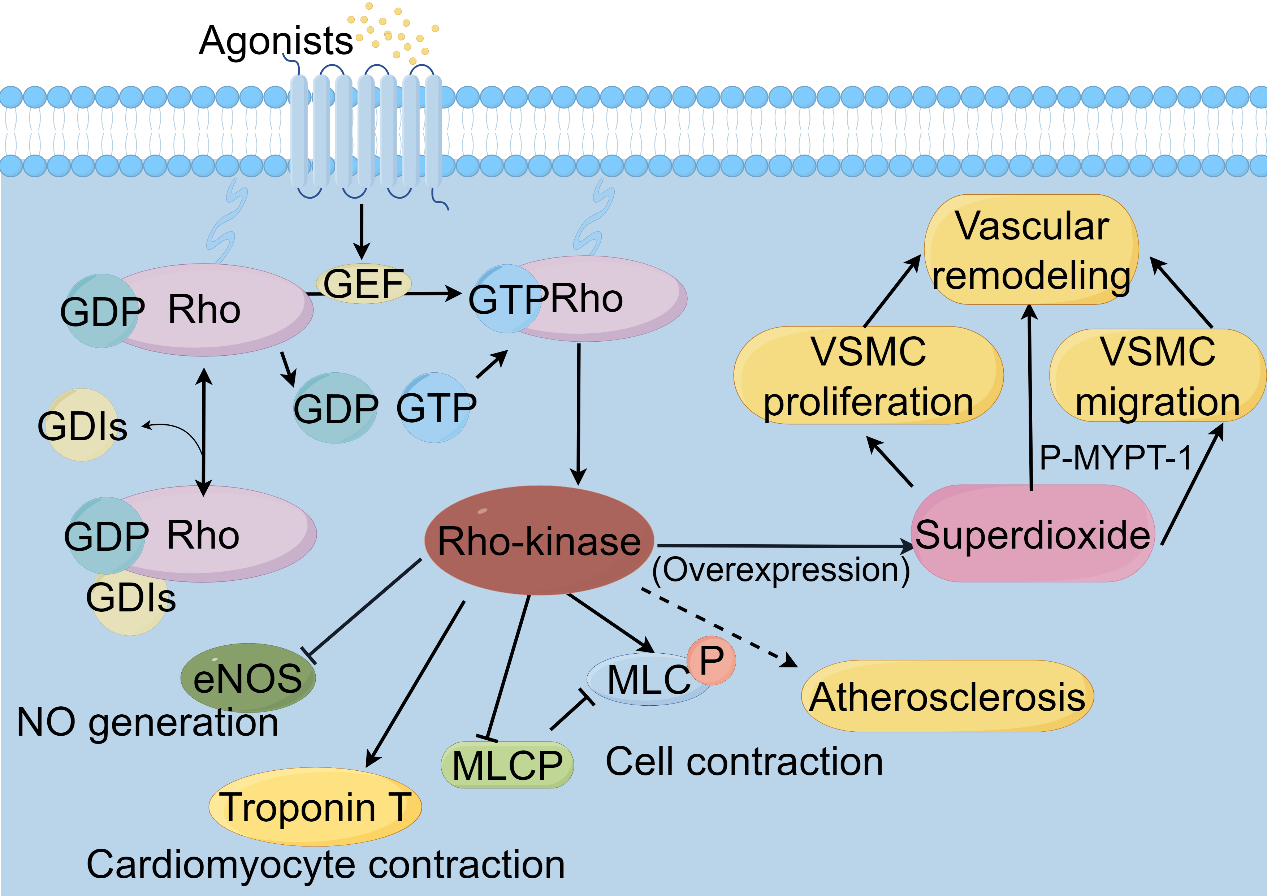
**

**Supplementary Fig. 5. RhoA/Rho kinase pathway.** Under physiological conditions, RhoA/Rho kinase maintains basal vascular tone equilibrium. However, upon vasoconstrictive stimuli, RhoA activation stimulates Rho kinase, leading to increased myosin light chain phosphorylation, actin cytoskeleton reorganization, and smooth muscle contraction, resulting in enhanced vasoconstriction. Pathological hyperactivation of this pathway contributes to endothelial dysfunction, vascular remodeling, and atherosclerosis, which are precursors to coronary microvascular dysfunction (CMD). Elevated RhoA/Rho kinase activity is associated with reduced NO synthesis and increased ET-1 production, disrupting vasomotor balance. Clinically, enhanced Rho kinase activity correlates with impaired myocardial perfusion in coronary artery disease, independent of significant coronary artery stenosis.

**
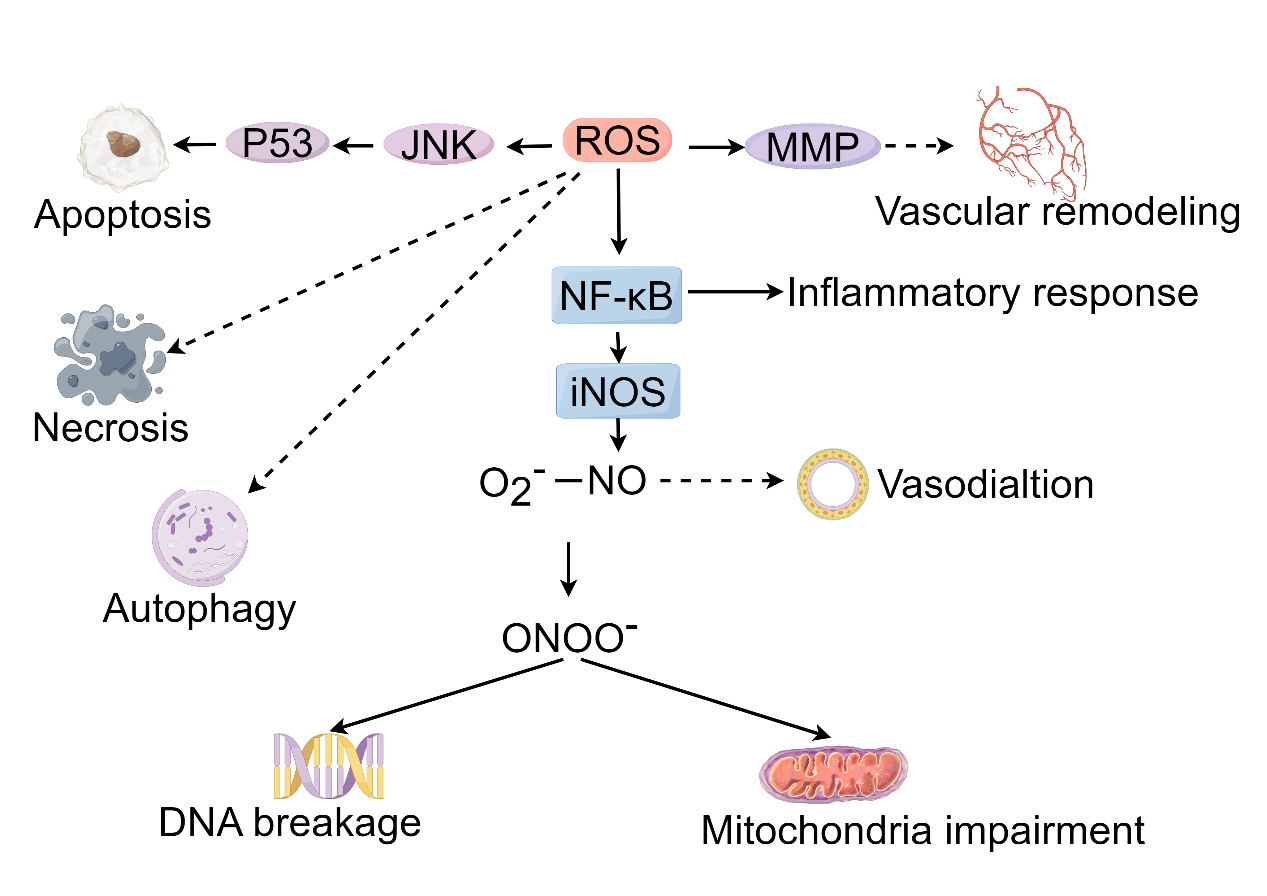
**

**Supplementary Fig. 6. Reactive oxygen species.** ROS influence coronary microvascular tone by modulating NO bioavailability. Excessive ROS react with NO to form peroxynitrite (ONOO-), diminishing NO levels and thus promoting endothelial dysfunction characterized by impaired vasodilation and enhanced vasoconstriction. This interplay is critical in CMD pathogenesis, where ROS-induced oxidative stress leads to endothelial apoptosis and mitochondrial dysregulation, contributing to myocardial ischemia-reperfusion injury. Moreover, ROS-mediated activation of matrix metalloproteinases (MMPs) drives vascular remodeling, with intimal thickening and fibrosis, narrowing the vascular lumen. Endothelial cell apoptosis, triggered by ROS, disrupts the endothelial barrier, potentiating atherosclerotic lesion formation. ROS also initiate an inflammatory response through the activation of NF-κB, escalating the transcription of pro-inflammatory cytokines and promoting leukocyte adhesion.

**
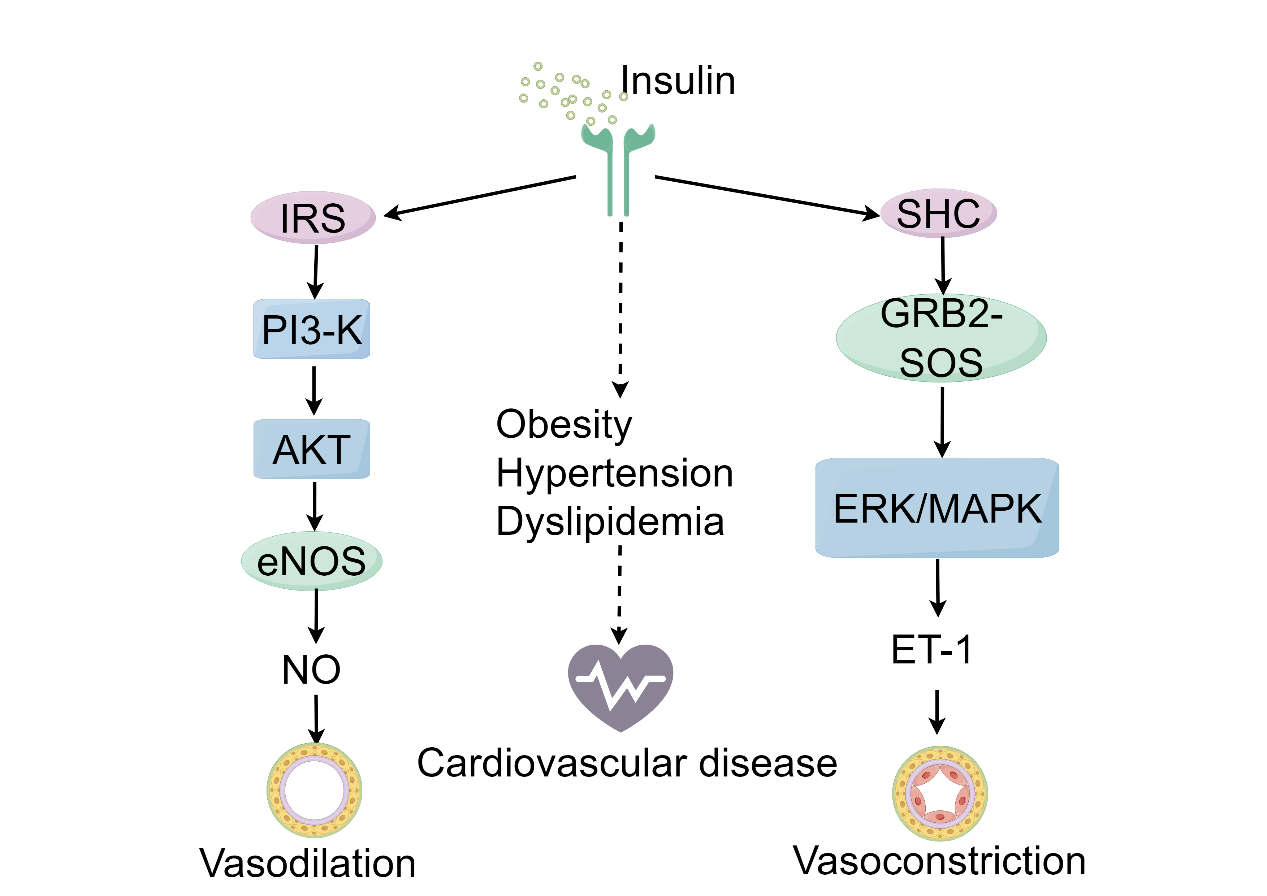
**

**Supplementary Fig. 7. Insulin signaling pathway.** Endothelial insulin signaling catalyzes NO production via the PI3K/Akt pathway, where insulin-mediated Akt phosphorylation activates eNOS, leading to vasodilation. Conversely, insulin can promote vasoconstriction via the MAPK pathway by stimulating ET-1 production, with both vasodilatory and vasoconstrictive influences typically achieving vascular homeostasis. The pathway for glucose metabolism becomes less responsive, while the MAPK-dependent vasoconstrictive response may be preserved or amplified, skewing the balance toward vasoconstriction, which can diminish coronary blood flow and contribute to CMD.

**
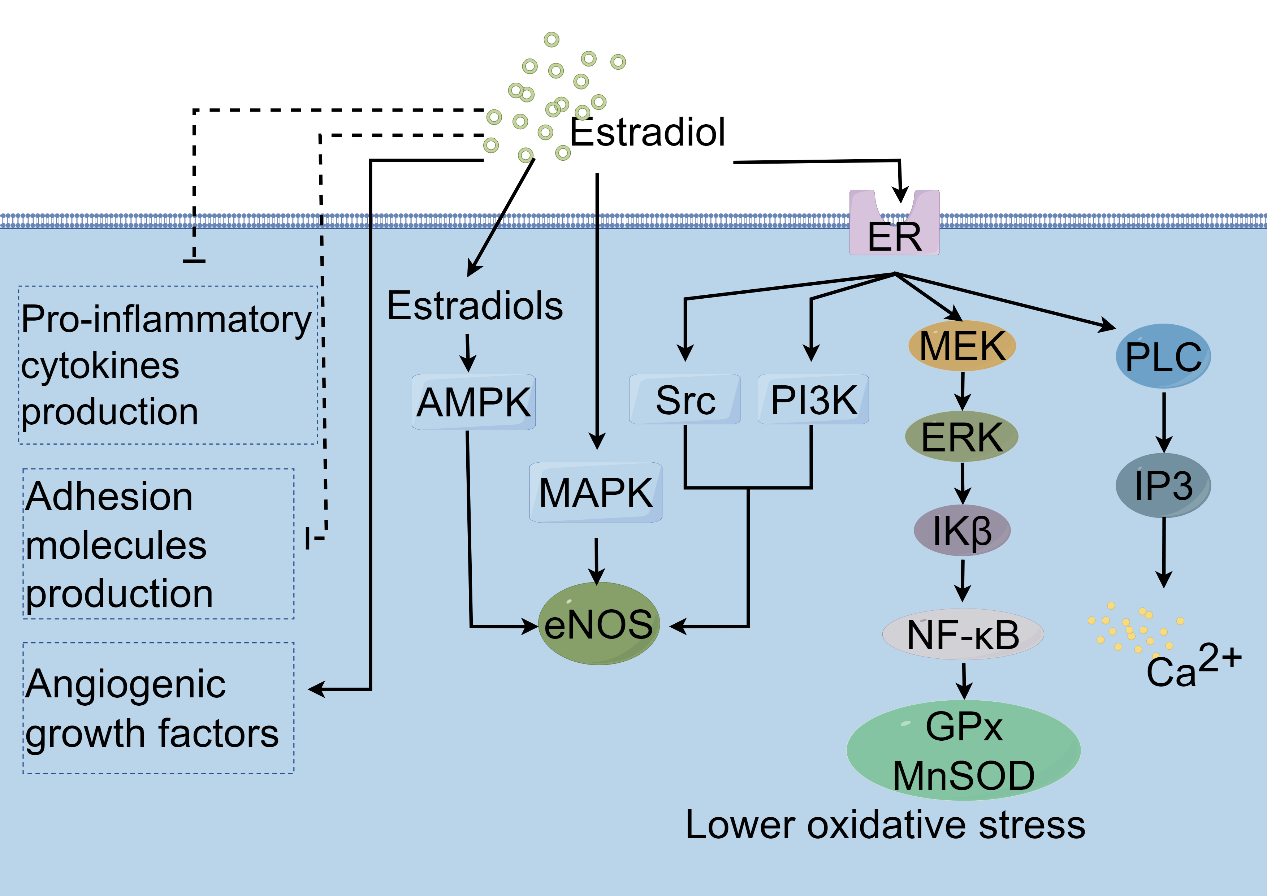
**

**Supplementary Fig. 8. Estrogen signaling pathway.** Estrogen modulates cardiovascular function through interactions with estrogen receptors alpha (ERα) and beta (ERβ), which, upon estrogen binding, translocate to the nucleus and alter gene transcription relevant to vascular homeostasis. Estrogen facilitates vasodilation, primarily by upregulating eNOS activity, increasing NO production, and attenuating vasoconstrictive responses to agents like angiotensin II (Ang II) and norepinephrine. Moreover, estrogen exerts anti-inflammatory effects, evidenced by downregulated expression of adhesion molecules and reduced cytokine-mediated leukocyte recruitment to the vascular wall. Its antioxidative role is highlighted by the upregulation of antioxidant enzymes and the suppression of ROS formation and lipid peroxidation, collectively safeguarding endothelial function. Estrogen also enhances mitochondrial efficiency in microvascular endothelial cells, optimizing calcium handling, ATP production, and attenuating radical generation. Furthermore, estrogen promotes microvascular angiogenesis by upregulating pro-angiogenic factors such as vascular endothelial growth factor (VEGF) and basic fibroblast growth factor (bFGF), fostering new vessel formation and thus supporting microvascular integrity.
